# Raman active diyne-girder conformationally constrained p53 stapled peptides bind to MDM2 for visualisation without fluorophores†

**DOI:** 10.1039/d4cb00288a

**Published:** 2025-01-15

**Authors:** Danielle C. Morgan, Laura McDougall, Astrid Knuhtsen, Lori Buetow, Craig F. Steven, Oscar A. Shepperson, Danny T. Huang, Alison N. Hulme, Andrew G. Jamieson

**Affiliations:** a School of Chemistry, Advanced Research Centre, University of Glasgow 11 Chapel Lane Glasgow G11 6EW UK andrew.jamieson.2@glasgow.ac.uk; b Cancer Research UK Scotland Institute, Garscube Estate Switchback Road Glasgow G61 1BD UK; c EaStCHEM School of Chemistry, The University of Edinburgh West Mains Road Edinburgh EH9 3JJ UK; d School of Cancer Sciences, University of Glasgow Glasgow G61 1QH UK

## Abstract

Peptide stapling is an effective strategy to stabilise α-helical peptides, enhancing their bioactive conformation and improving physiochemical properties. In this study, we apply our novel diyne-girder stapling approach to the MDM2/MDMX α-helical binding region of the p53 transactivation domain. By incorporation of an unnatural amino acid to create an optimal *i*, *i* + 7 bridge length, we developed a highly α-helical stapled peptide, 4, confirmed *via* circular dichroism. This diyne-girder-stapled peptide demonstrated enhanced helicity and nanomolar binding affinity for MDM2, as assessed by fluorescence polarisation. Crucially, peptide 4 exhibited strong selectivity for MDM2, with approximately 100-fold reduced affinity for MDMX. Molecular modeling and docking studies suggested that this selectivity arose from diminished hydrophobic interactions at the MDMX binding site, driven by the diyne-girder's constrained geometry. The use of the diyne-girder, a unique feature amongst stapled peptide analogues, for cellular visualisation using Raman spectroscopy in the “cell-silent” region was explored. This capability potentially offers a novel method for tracking stapled peptides in biological systems without the need for large fluorophores. Overall, peptide 4 represents a promising tool for probing MDM2 activity and a valuable addition to the arsenal of peptide-based therapeutic strategies.

## Abstract 2

This work describes the development of Raman-active diyne-girder stapled peptides that selectively bind MDM2 and enable cellular visualisation without fluorophores, providing a new method for studying protein–protein interactions.

## Graphical abstract

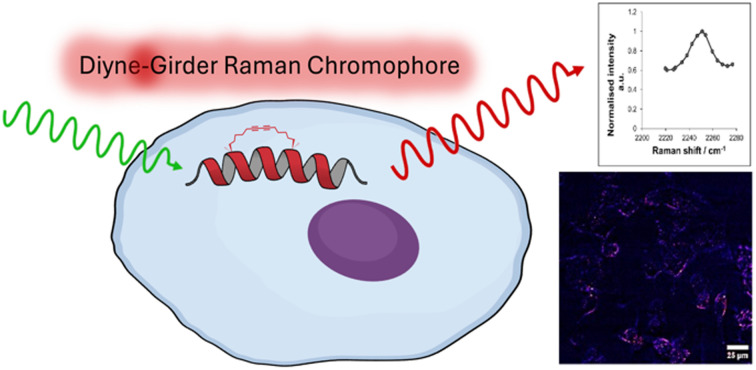

## Introduction

**Fig. 1:**
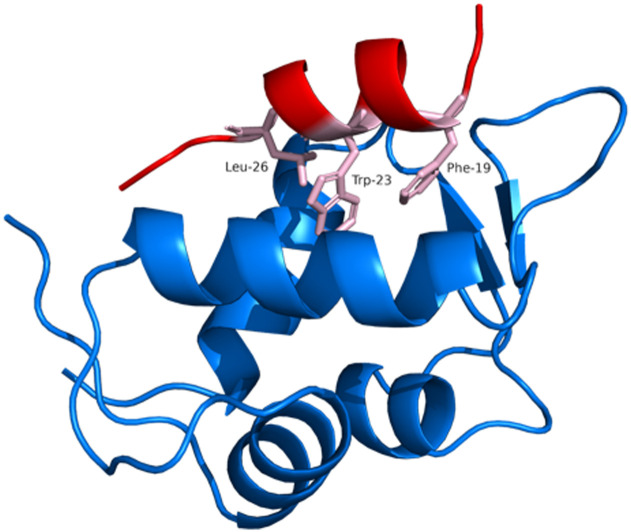
Crystal structure of MDM2 protein (blue) bound to the α-helix comprised of residues 16–29 of p53 (red). The “hotspot” residues in p53 are represented as sticks (pink) (PDB: 1YCR).^4^

Mouse double minute 2/X homologs (MDM2/MDMX) are the primary negative regulators of the p53 tumour suppressor.^1^ The p53 suppressor plays a critical role in protecting cells from malignant transformation by inducing cell-cycle arrest and apoptosis in response to DNA damage and cellular stress.^1^ MDM2/MDMX forms a protein–protein interaction (PPI) with the transactivation domain of p53, resulting in a complex allowing identification of this protein for degradation.^2,3^ Specifically, wild-type (WT) p53 features an N-terminal α-helical portion that interacts with MDM2/MDMX. This interaction relies heavily on specific residues, namely Phe^19^, Trp^23^, and Leu^26^, identified using the p53–MDM2 crystal structure (PDB: 1YCR).^4^ The key residues are arranged on a single face of the α-helix of p53 and make a substantial contribution to the binding energy (Fig. 1).^4^ The identification of the p53 sequence that interacts with MDM2 has facilitated the development of small molecules and peptides aimed at disrupting the p53–MDM2 PPI as a strategy to restore p53-dependent tumour suppressor activity in cancers.

A reported strategy for designing therapeutics to disrupt this specific PPI involves the use of α-helical stapled peptides.^5,6^ One of the initial key developments in this field included such stapling, with work from Bernal *et al.* (2005), whereby they reported ‘stabilised α-helical-8’ (SAH-8), a peptide derived from WT p53, that binds to MDM2/MDMX and activates the p53 response in cells.^7^ The co-crystal structure of SAH-8 in complex with MDM2 provided greater insight into the mechanism of binding, specifically identifying a hydrophobic pocket on the MDM2 surface where p53 engages in numerous hydrophobic interactions.^7^

Carvajal *et al.* (2018) advanced the development of stapled peptides to disrupt the p53–MDM2 interaction by specifically targeting the core binding residues (Phe^19^, Trp^23^, and Leu^26^).^4,8^ Through iterative optimisation studies of the linear α-helical fragment of p53, several residues were modified, leading to enhanced biological potency. Of most interest was the replacement of Leu^26^ with the unnatural amino acid, cyclobutylalanine (Cba), enhancing the hydrophobic interaction within the pocket, initially brought to light by Bernal *et al.* (2005).^7^ The work culminated in a progenitor of the initial ATSP-7041 peptide, ALRN-6924, recognised as the first cell-permeating, stapled α-helical peptide to progress into clinical development.^9,10^ ALRN-6924 progressed to phase II clinical trials (phase I – NCT02909972 and phase II – NCT02264613), as a dual inhibitor of MDM2/MDMX for patients with solid tumour and lymphomas bearing WT Tp53.^11,12^

As has been previously evidenced by the success of stapled analogues to inhibit the p53–MDM2 interaction, hydrocarbon alkene stapling is a powerful tool in the chemical biologist's toolbox.^10,13,14^ However, despite the impressive success of this approach a number of limitations remain – namely, the requirement of two non-native alkene amino acids, a ruthenium-based Grubbs catalyst, and resultant production of a mixture of *cis*- and *trans*-isomers with *i*, *i* + 7 alkene stapling.^15,16^ In addition, to study alkene stapled peptides within cellular environments, a typically large and hydrophobic fluorophore is required to adequately visualise the peptide target.^17,18^

We recently reported a novel type of diyne-girder peptide stapling strategy designed to overcome the limitations of hydrocarbon alkene stapling.^19^ This strategy provides stable, α-helical peptidomimetics incorporating a Raman active conformational constraint, removing the requirement of any additional fluorophores for visualisation within cellular environments.^13,19^ Our work presented herein investigates the application of our diyne-girder stapling strategy to the p53 peptide. Using this new chemical methodology, we synthesised a series of Raman-active diyne-girder stapled p53 peptides. Our peptides displayed enhanced helicity, and, in a fluorescence polarisation binding assay, we observed a preference for binding to MDM2 over MDMX. With the help of molecular docking, we were able to elucidate the underlying molecular mechanism of this specificity for MDM2 over MDMX.

Our work presented herein sheds new light on the potential of a recently developed, isomerically pure, hydrocarbon constraint for inhibiting the MDM2–p53 PPI interaction. Furthermore, we uncover previously unknown details regarding the binding pocket of MDMX and how it varies from MDM2. Our work culminates in employing this hydrocarbon constraint with coherent Raman microscopy to visualise our constrained peptide intracellularly.

## Results and discussion

**Fig. 2:**
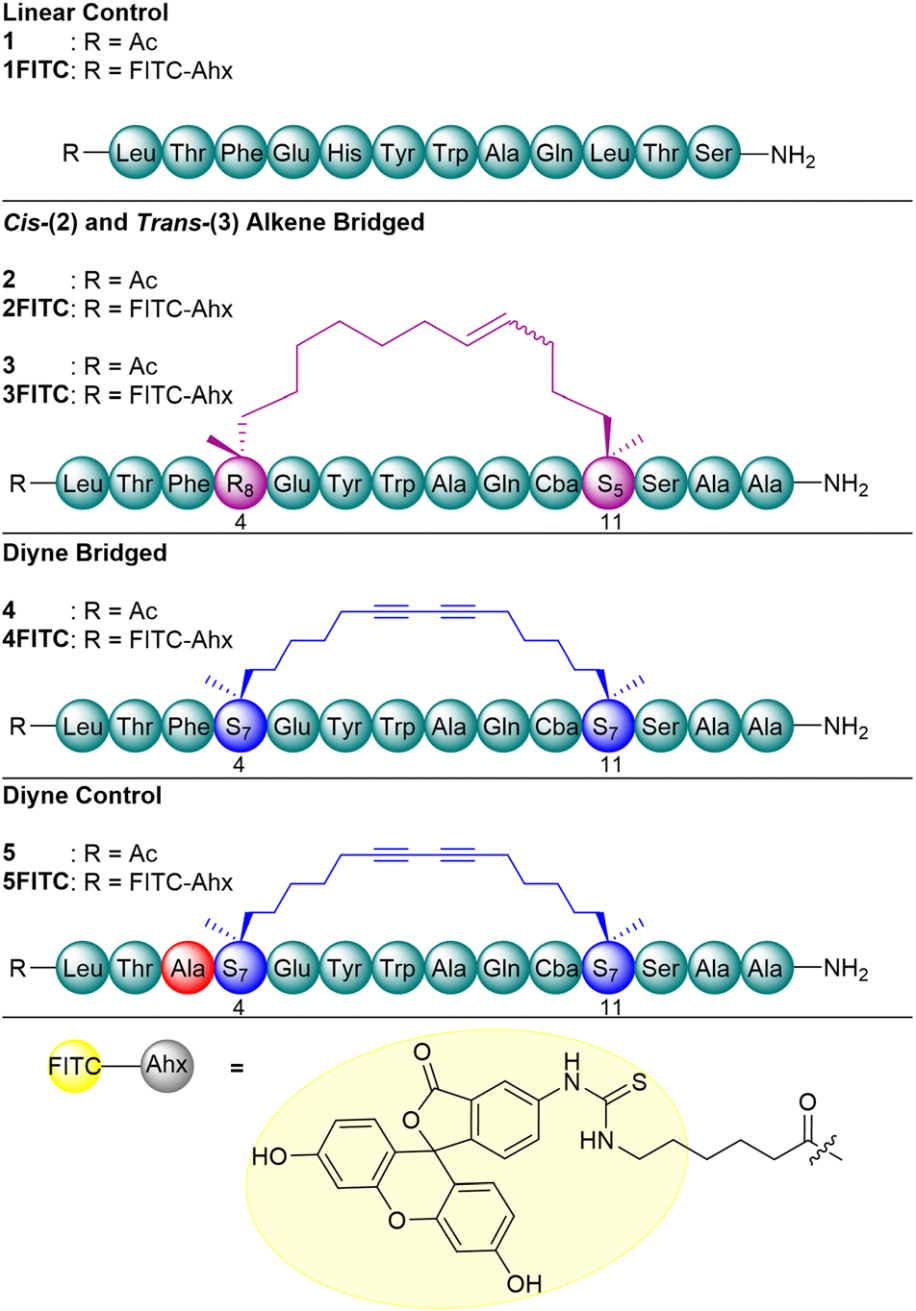
p53 (15–29) peptide analogues. Native peptide (green), alkene staple (purple), diyne staple (blue), amino acid substitution (red), spacer amino acid (grey) and fluorescent tag (yellow) are highlighted.

We envisaged the synthesis of an *i*, *i* + 7 diyne-girder stapled peptide analogue, 4, of the extensively studied, alkene stapled peptide, ATSP-7041 (2/3) (Fig. 2).^19,20^ Alongside the synthesis of our diyne-girder analogue, 4, we sought to synthesise the linear control peptide, ATSP-3848 (1), and a diyne control peptide (5, Phe^3^ to Ala^3^) for side-by-side comparison of MDM2/MDMX binding affinity (Fig. 2).^20^ For each peptide prepared herein, the N-terminus was either acetylated (1–5) or fluorescently labelled (1FITC–5FITC) with fluorescein isothiocyanate (FITC) for biological visualisation by fluorescence polarisation and microscopy.

### Fmoc-solid phase synthesis (SPPS) of stapled peptide analogues

Peptides were prepared by microwave-assisted 9-fluorenylmethyloxycarbonyl (Fmoc)-SPPS.^21^ ChemMatrix© Rink-amide resin (0.45 mmol g^−1^) was employed to achieve C-terminal carboxamide functionality.^22^ Coupling reactions were achieved using Fmoc protected amino acids (5 equiv.), OxymaPure (5 equiv.) and *N*,*N*′-diisopropylcarbodiimide (DIC) (5 equiv.) in dimethylformamide (DMF) (90 °C, 2 min, 55 μW). Where unnatural amino acids ((*R*)-2-((((9*H*-fluoren-9-yl)methoxy)carbonyl)amino)-2-methyldec-9-enoic acid [Fig. 2, R_8_], (*S*)-2-((((9*H*-fluoren-9-yl)methoxy)carbonyl)amino)-2-methylhept-6-enoic acid [Fig. 2, S_5_] and (*S*)-2-((((9*H*-Fluoren-9-yl)methoxy)carbonyl)amino)-2-methylnon-8-ynoic acid [Fig. 2, S_7_]) were employed, couplings were repeated, and reaction time doubled (90 °C, 2 × 4 min, 55 μW). Deprotection of N-terminal Fmoc protected amino acids was achieved using 20% morpholine (v/v) in DMF (90 °C, 1 min). The resin was washed thoroughly between reactions using DMF. For acetylated peptide analogues the N-terminal acetylation was achieved with acetic anhydride (5 M) and diisopropylethylamine (DIPEA) (5 equiv.) in DMF (r.t., 10 min). For the fluorescently labelled counterparts, Fmoc-6-aminohexanoic acid (Fmoc-Ahx) was first coupled as a spacer using identical reaction conditions to those employed for standard amino acids.^23^ Following removal of Fmoc from Ahx, FITC (2 equiv.) was coupled to the free amine using DIPEA (8 equiv.) in DMF (r.t., 16 h, excluding light). It should be noted that for fluorescently labelled stapled peptide analogues (1FITC–5FITC), the coupling of the fluorescein tag was performed following macrocyclisation to form the staple functionality.^24^

To achieve the linear control peptide, 1, the linear peptidyl resin underwent cleavage from the resin, with concomitant global deprotection using trifluoroacetic acid (TFA)/triisopropylsilane (TIPS)/H_2_O (95/2.5/2.5, v/v/v) over the course of 3 h at r.t.

To achieve the *i*, *i* + 7 hydrocarbon alkene stapled peptides, *cis*-2 and *trans*-3, the linear peptide was synthesised with R_8_ (at position 4) and S_5_ (at position 11). Cyclisation was performed by Ring-Closing Metathesis (RCM) using Grubbs catalyst (excluding light).^15^ The reaction was confirmed as complete by cleavage of a small portion of the peptidyl resin and subsequent analysis by RP-HPLC and LC-MS. As previously alluded to, RCM reactions are non-stereoselective, resulting in a mixture of *cis*- (*cis*-2) and *trans*-alkene (*trans*-3) products (Fig. 2). These isomers were isolated as the mixtures of products following resin cleavage and concomitant global deprotection by TFA/TIPS/H_2_O (95/2.5/2.5, v/v/v) over the course of 3 h at r.t. The resultant mixture of isomeric peptidyl products (*cis*-2 and *trans*-3) was purified by preparative RP-HPLC (yields = 4%, *cis*-2; 2%, *trans*-3).

**Scheme 1:**
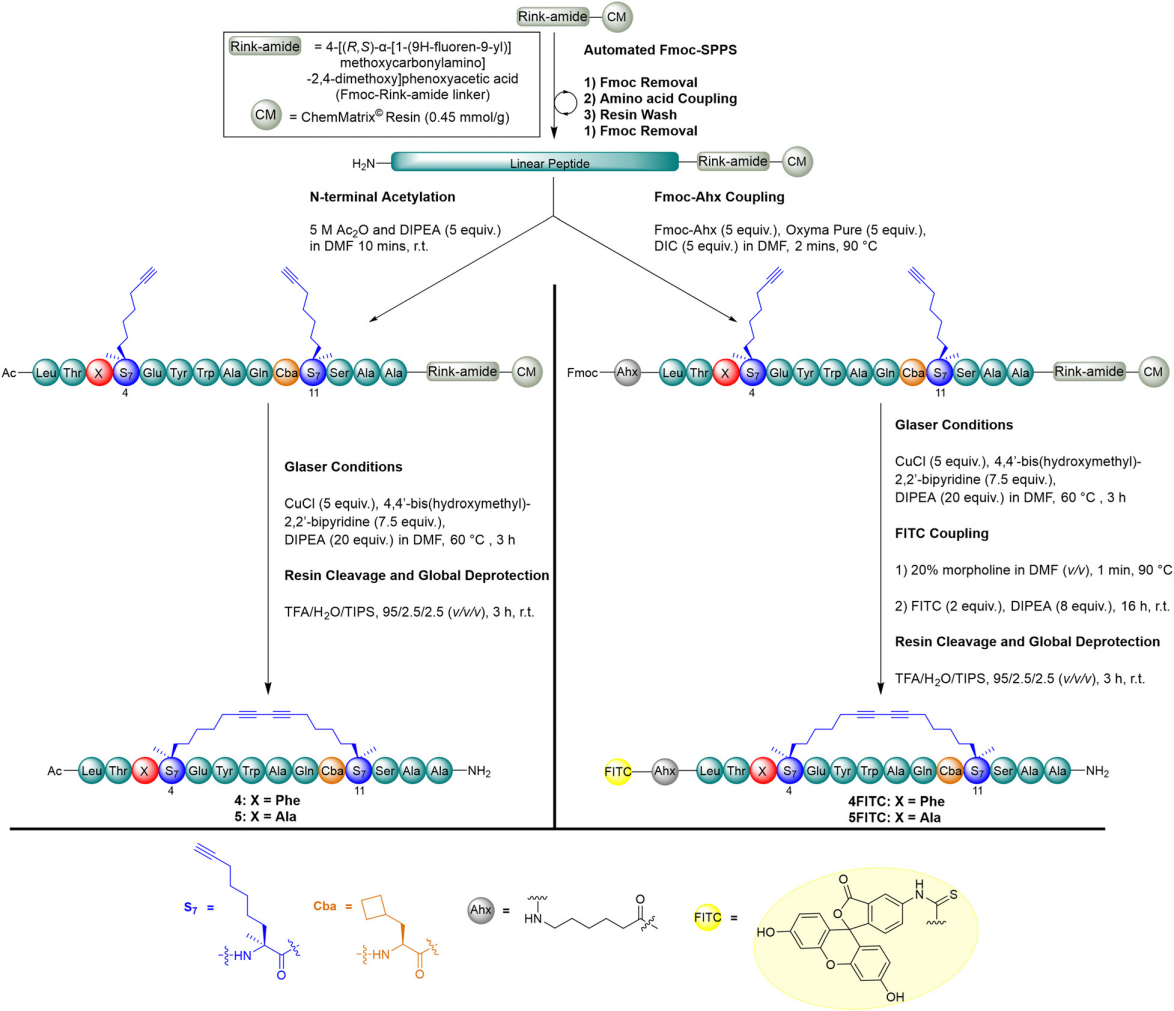
Synthetic protocol for the synthesis of diyne stapled peptides 4 and 5, and FITC tagged peptides, 4FITC and 5FITC. Key components shown for diyne residues and bridges (blue), unnatural amino acids (orange), fluorescent tag (yellow) and variable amino acid for synthesis of either 4/4FITC or 5/5FITC (red, X).

To achieve the synthesis of the *i*, *i* + 7 diyne-girder stapled peptides, 4 and 5, the linear peptides were prepared with S_7_ at positions 4 and 11 (Scheme 1).^19^ Macrocyclisation to produce the diyne conformational constraint was achieved on-resin, using Glaser reaction conditions. Conversion to the diyne-girder stapled product was determined by examination of analytical RP-HPLC and LC–MS following cleavage of a small portion of the peptidyl resin. Upon reaction completion to the desired single product, resin cleavage and concomitant global deprotection preceded purification by preparative RP-HPLC to provide the desired diyne-girder peptides, 4 and 5, in excellent yields (10% and 11%).

All final peptides were purified to a purity greater than 97% (Table S1 and Fig. S1–S30, ESI†).

### Conformational analysis by circular dichroism (CD) spectroscopy

Following the successful synthesis of the diyne peptide 4, it was important to identify the effect of the modified staple on the α-helical nature of the peptide. The effectiveness of the diyne staple as an α-helical constraint was assessed by CD. Experiments were set out to directly compare the diyne stapled peptide 4 to the alkene stapled peptides (*cis*-2 and *trans*-3), with the linear peptide 1 employed as a control. CD was performed at two concentrations to investigate whether peptide concentration influenced helicity, as coiled-coil structures are known to increase helicity.^25^ Peptide concentrations were quantified using a NanoDrop™ One Microvolume UV-Vis spectrophotometer using the UV absorption of the peptides at 280 nm.^26^ Extinction coefficients of the peptides were calculated from Trp and Tyr residues and the Beer–Lambert law employed to calculate the concentrations.

**Fig. 3:**
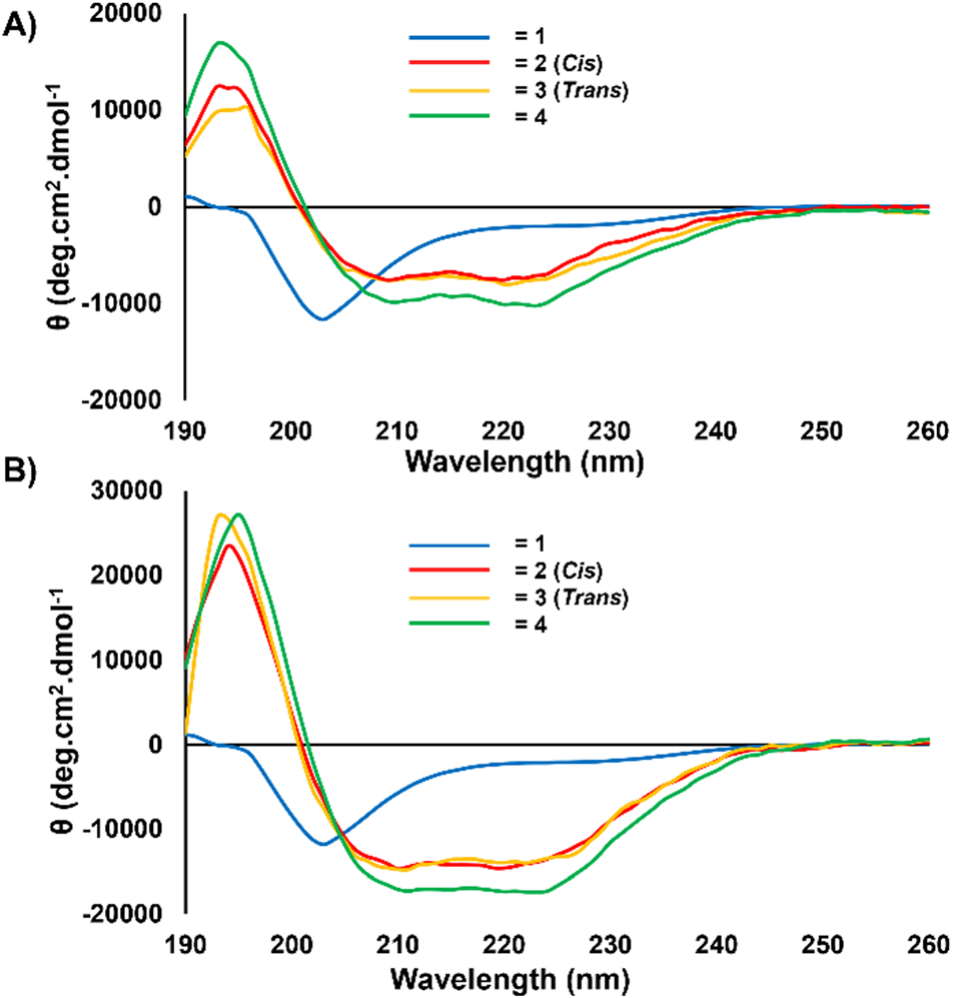
CD of peptides 1–4 at varying concentrations in PBS (pH 7.4), (A) 25 μM and (B) 50 μM. α-Helical negative maxima are observed for peptides 2–4 at 208 and 222 nm.

Each of the peptides (1–4) were analysed at 25 μM and 50 μM in phosphate buffered saline (PBS, pH 7.4) (Fig. 3). CD spectra were measured from 190–260 nm and the characteristic α-helical negative maxima examined for their intensity at 208 nm and 222 nm.^27^ The CD experiments confirmed that the diyne (4) exhibits similar characteristic α-helical negative maxima as both alkene isomers (*cis*-2 and *trans*-3). The three stapled peptides (2–4) all exhibited spectra of far more ordered nature than the linear control peptide (1). This is to be expected due to the random coil nature of 1. Interestingly, the diyne stapled peptide, 4, exhibited a slightly enhanced helicity when compared to the alkenes (*cis*-2 and *trans*-3), suggesting that the diyne staple may be a more effective α-helical conformational constraint than the alkene staple for this peptide. Helicity calculations were performed following conversion of the measured degrees of ellipticity (*θ*) data to mean residue ellipticity (MRE) (eqn (S1), ESI†). Percentage helicities were calculated from the MRE values observed at 222 nm for the stapled peptide (2–4) with those equations as defined by Luo and Baldwin (eqn (S2), ESI†).^28^ The diyne-girder stapled peptide 4 was the most α-helical with a helicity of 59%. In comparison, the *cis*/*trans*-isomers of the alkene stapled peptides (2/3) both exhibited a reduced percentage helicity of 48%. As such, the effect of the diyne conformational constraint when applied to this peptide, is to enhance induction of the α-helical bioactive conformation.

### Fluorescence polarisation (FP)

**Fig. 4:**
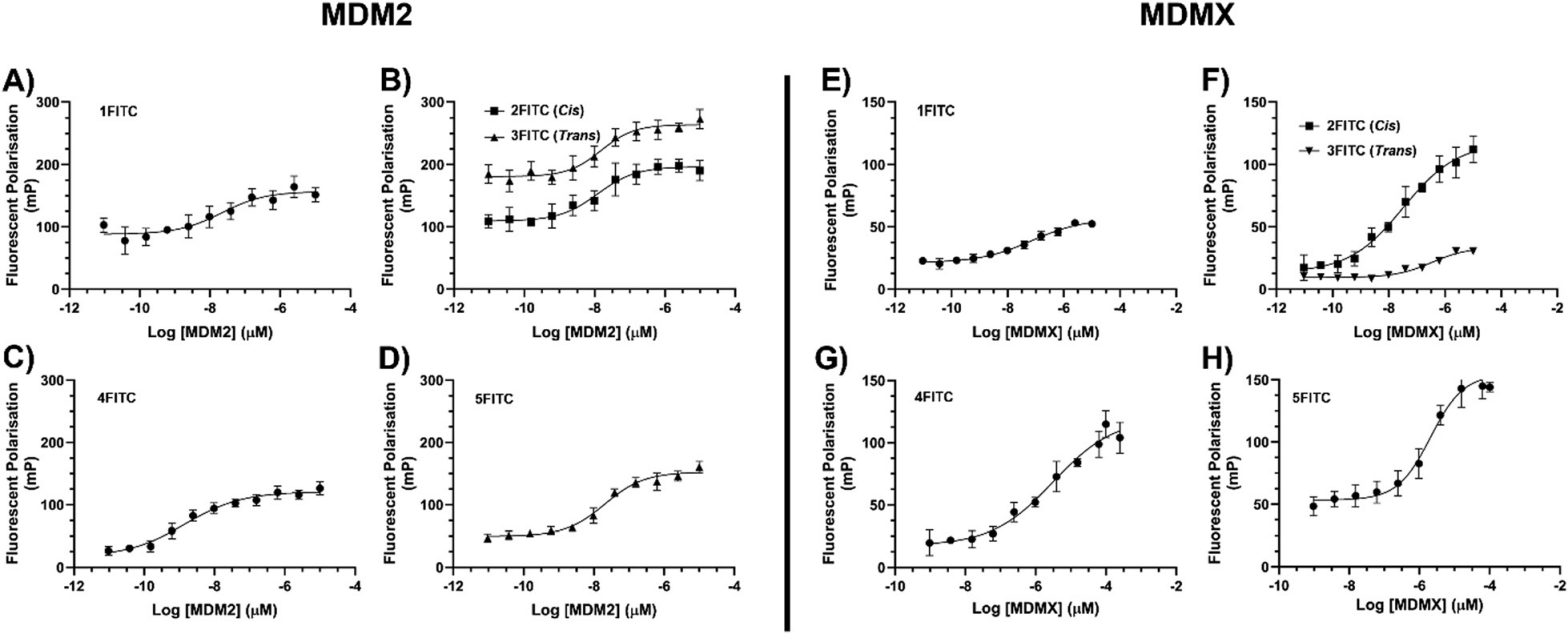
FP binding curves of fluorescently labelled peptides (1FITC–5FITC) to MDM2 (A)–(D) and MDMX (E)–(H). Error bars = 2 SD, (*n* = 3).

The binding properties of the peptide were assessed using a biophysical FP assay. Specifically, a direct binding assay was conducted to calculate the *K*_d_ values of the fluorescent peptide ligands binding to the target proteins (eqn (S3), ESI†).^29^ The fluorescently labelled peptides (1FITC–5FITC) were tested for binding to MDM2 (1–138) and MDMX (1–134). The peptides were first tested for binding with MDM2 (1–138). Since the linear (1FITC) and alkene-stapled peptides (2FITC/3FITC) had previously demonstrated binding in the low nM range (as measured by SPR and FP), an FP assay was conducted under comparable conditions to those reported in the literature.^20^ Peptide solutions were made up to a concentration of 10 nM and varying concentrations of protein (10 μM to 9 pM) were incubated with the peptide with FP measurements taken after 3 h (Fig. 4). The FP binding data confirmed the low nM binding of 1FITC–3FITC as reported by Chang *et al.* (2013) (Table 1).^20^ It was pleasing to observe that the diyne stapled peptide 4FITC also exhibited low nM binding affinity for MDM2 (Table 1). The final analogue examined for its binding of MDM2 was 5FITC, the diyne negative control. The single amino acid substitution of Phe^3^ to Ala^3^ was expected to disrupt binding, and this feature was observed with 5FITC exhibiting a greater than 10^3^-fold reduction in *K*_d_ for MDM2.

**Table 1:** FP *K*_d_ (nM) for linear and stapled analogues, 1FITC–5FITC to MDM2 (1–138) and MDMX (1–134)

Peptide	*K*_d_ (nM) MDM2	*K*_d_ (nM) MDMX
1FITC (ATSP-3848)	1.49 ± 0.29	20.1 ± 0.23
*cis*-2FITC (ATSP-7041)	0.33 ± 0.19	8.42 ± 0.09
*trans*-3FITC (ATSP-7041)	0.31 ± 0.06	7.86 ± 0.15
4FITC	1.39 ± 0.08	203 ± 54
5FITC	3680 ± 151	1120 ± 252

Due to alkene stapled peptides being reported as dual MDM2/MDMX ligands we also investigated the potential for our diyne analogue (4FITC) to act as a dual ligand of MDM2/MDMX.^9,10^ The fluorescently labelled peptides (1FITC–5FITC) were tested against MDMX (1–134) in an identical fashion to that employed for MDM2. Examination of the data (Fig. 4 and Table 1) for the peptides tested confirms the low nM binding of the linear peptide (1FITC) and the alkene stapled peptides (*cis*-2FITC and *trans*-3FITC), as reported by Chang *et al.* (2013).^20^ However, of paramount importance to us, was the 100-fold decrease in binding for MDMX exhibited by our diyne stapled analogue (4FITC). The variance in binding for our diyne stapled peptide (4) between MDM2 and MDMX suggested that the difference in the binding pocket of these similar proteins may require more investigation, with molecular modelling the preferred tool to facilitate such investigation.

### Structural analysis of MDM2/MDMX using molecular modelling

Peptide stapling can have the effect of inducing the α-helical bioactive conformation of peptides, thereby paying the entropic penalty of folding and thus enhancing binding affinity. Previously reported work has suggested that there is a correlation between binding affinity and α-helicity.^30^ This led to the question as to why 4FITC exhibited a reduction in dual affinity despite having greater α-helicity (*vide supra*). We proposed the answer may lie within the nature of the diyne staple, and further, that it may interact less favourably with the binding pocket of MDMX than MDM2. Molecular modelling is a widely utilised technique for exploring the interactions and binding affinities between proteins and peptides. We thus sought to employ molecular modelling to understand the nature of molecular recognition of our diyne-girder stapled peptides with MDM2/MDMX. Computational software (molecular operating environment (MOE)) was employed to model the PPI between the stapled peptides and MDM2/MDMX.^31^ Initially, we examined the surface topology and amino acid sequences of the p53 binding site on MDM2 and MDMX to identify differences that could explain the selectivity of the diyne-girder stapled peptide (Fig. S31, ESI†). Overlaying the two proteins revealed a single variable amino acid (Phe^55^ for MDM2 and His^55^ for MDMX) in the binding pocket (Fig. S31, ESI†). The location of this amino acid modifies the surface of the binding pocket exactly where the hydrocarbon staple is positioned during binding. Hydrocarbon stapled analogue ATSP-7041 (*trans*-(**3**)) is known, from the crystal structure, to form hydrophobic interactions with the protein through the alkene hydrocarbon constraint.^20^ We hypothesised that the reason behind the decreased binding affinity for our diyne-girder stapled peptide over the alkene stapled peptide is likely due to the decreased torsional flexibility of the diyne-girder conformational constraint. To investigate this effect further we performed a series of docking experiments.

### Docking experiments of stapled peptides to MDM2/MDMX

**Fig. 5:**
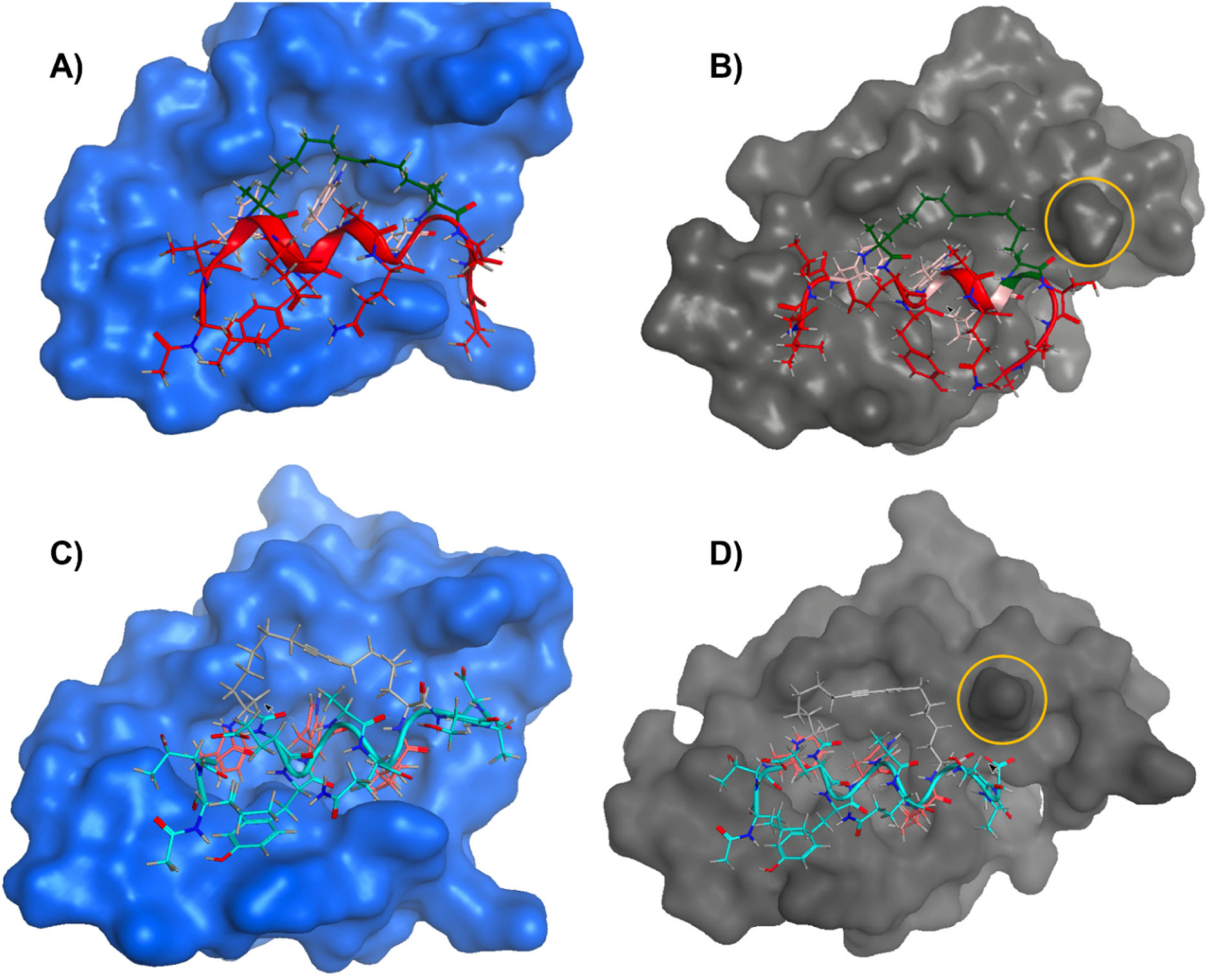
Molecular models (front views) showing an overlay of the binding of alkene stapled peptide *trans*-3 (red) and diyne-girder stapled peptide 4 (cyan) to the binding pockets of MDM2 (blue, A/C) and MDMX (grey, B/D). Key elements include the alkene hydrocarbon staple (dark green), the diyne staple (grey), the key hydrophobic binding residues Phe, Trp and Cba (pink), and the point mutation that modifies the binding pocket of MDMX (yellow circle). Molecular models were produced using MOE.^30^

Docking experiments between the stapled peptides and MDM2/MDMX (PDB: 1YCR & 4N5T) were conducted using MOE to predict the position of the hydrocarbon chains of *trans*-3 and diyne 4 (Fig. 5 and Fig. S32–S35, ESI†).^31^ The resulting docking experiments clearly evidenced the alkene staple (Fig. 5(A) and (B), green) of 3 making key hydrophobic interactions and fitting well into the binding pocket of both MDM2 (Fig. 5(A)) and MDMX (Fig. 5(B)). The key peptide binding residues Phe, Trp and Cba (Fig. 5, pink) were observed making hydrophobic interactions with the surface of the protein binding pocket, consistent with alkene hydrocarbon bridged analogues in literature.^4,7^ The resulting docking scores (Fig. S32–S35, ESI† S, more negative values are more favourable) provided by MOE suggest that binding of alkene stapled peptide 3 to both MDM2 and MDMX is favourable and similar for both proteins. On the contrary, MOE docking simulations of the diyne-girder stapled peptide, 4, revealed significantly different results to those for alkene stapled peptide *trans*-3. For MDM2 (Fig. 5(C)), the diyne stapled peptide 4 (Fig. 5(C) and (D), grey) docks well into the pocket and the same interactions of the hydrophobic side-chains (Phe, Trp and Cba) were observed. However, upon examination of the diyne-girder stapled peptide binding to MDMX (Fig. 5(D)) we observed that the point mutation at residue 55 (from Phe to His) significantly impacts the surface topology of the protein next to the binding pocket. This point mutation for MDMX appears to reduce hydrophobic interactions of the diyne-girder constraint with the surface of the protein that are observed with alkene stapled peptide *trans*-3. The loss of hydrophobic interactions between the conformational constraint of diyne stapled peptide 4 on binding to MDMX provides an explanation for the distinct selective binding affinity, as judged by FP (Fig. 4 and Table 1), observed for diyne peptide, 4, to MDM2/MDMX.

### Conformational search on diyne stapled peptide 4

To further investigate our hypothesis, a conformational search was conducted on the diyne stapled peptide 4 to predict the conformational variability. One hundred conformations were generated using Trp^7^ as the point of ref. 4.

The resulting conformational search generated an overlay of all the possible conformations of the diyne peptide (Fig. S36, ESI†), each associated with varying Gibbs free energy (*G*) values. This analysis helped identify the most stable conformers by comparing their energy profiles, allowing for insights into the peptide's flexibility and preferred structural arrangements. The variations in *G* values reflect the peptide's conformational diversity and its potential to adopt multiple energetically favourable states. Examination of the possible conformations revealed high regions of homology for the peptide backbone sequence. Alternatively, the staple region showed a much greater variability of conformations. The greater degree of variability of the stapled regions suggests that the staple may indeed be impacting the binding of MDMX, consistent with our modelling data (*vide supra*).

### Cellular uptake experiments using fluorescence microscopy

To investigate and confirm the cellular uptake of the diyne-girder stapled peptide 4 compared to alkene stapled peptide *trans*-3, we employed fluorescence microscopy. Importantly, previous work has determined 3FITC to be equipotent in terms of both MDM2/MDMX binding and cellular potency to the non-fluorescently labelled peptide, *trans*-3.^20^ This indicates that the addition of FITC as a fluorophore to the N-terminus of the peptide does not significantly alter the biophysical/biological properties of these peptides. We therefore predicted, due to the identical natures of the peptides barring hydrocarbon conformational constraint, that peptide 4FITC would exhibit a similar maintenance of binding and potency to peptide 4.

HeLa cells were treated with 20 μM of the fluorescein labelled peptides, 1FITC–4FITC, Hoechst 33342 nuclear stain, and imaged 4.5 h post-treatment (Fig. S37, ESI†). 2FITC/3FITC and 4FITC all exhibited a diffuse intracellular localisation, confirming efficient cellular penetration. As expected, the native peptide 1FITC showed no cellular internalisation, providing strong evidence for the requirement of stapling as a tool for cellular penetration.

### Raman spectroscopy

Traditionally, cell-uptake experiments are performed using fluorescently labelled peptides.^32^ Fluorescent labels are often large hydrophobic groups and frequently alter peptide–target interactions.^17,18^ A conformational constraint (the diyne) that also acts as a chromophore for imaging would provide a unique advantage in circumventing the need for fluorophores.^19^

Following confirmation that activity is maintained for our diyne-girder stapled peptide 4 (*vide supra*), we sought to investigate the suitability of our diyne-girder staple as a Raman-active tool. Spontaneous Raman spectroscopy of diyne-girder stapled peptide 4 (as a lyophilised powder) was conducted and the resultant spectrum (Fig. S38, ESI†) of the diyne stapled peptide 4 evidenced a significant peak at ∼2252 cm^−1^. Crucially, this peak is observed in the “cell-silent” region (1800–2800 cm^−1^) of the spectrum.^33^ To compare the diyne-girder and alkene stapled peptides, Raman spectroscopy was also performed on the alkene stapled peptide, *trans*-3. As expected, the spectrum exhibited a complete absence of peaks in the “cell-silent” region for the alkene peptide *trans*-3 (Fig. S38, ESI†).

**Fig. 6:**
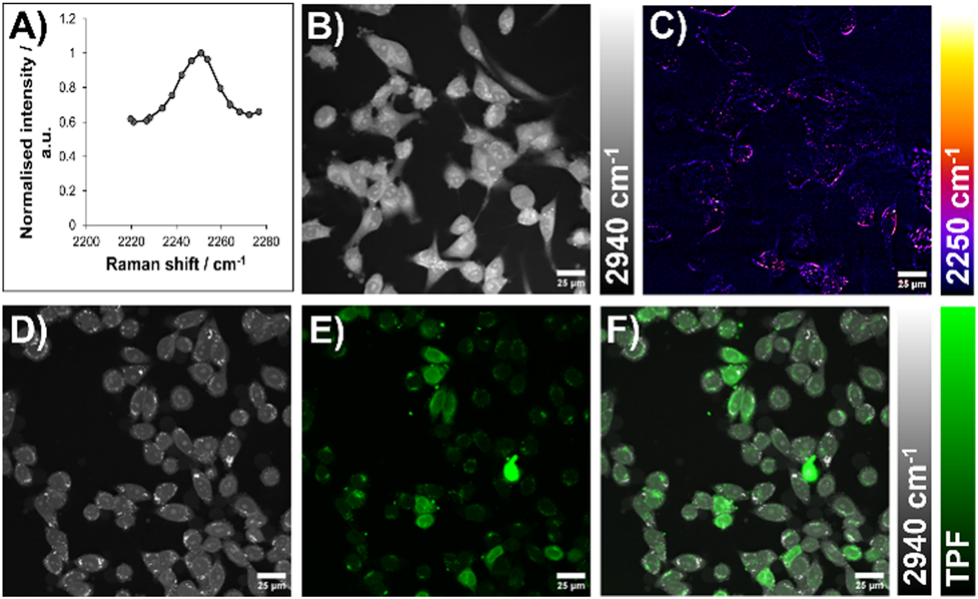
SRS imaging of 4 and 4FITC in live cells. (A) Hyperspectral SRS scan of 4 in solution (10 mM). (B) and (C) ES-2 cells incubated with 4 (100 μM, 3 h). (D)–(F) HeLa cells incubated with 4FITC (20 μM, 3 h). Contrast achieved by tuning to: (B) and (D) 2940 cm^−1^, CH_3_ (proteins); (C) 2250 cm^−1^, C≡C (in diyne-girder stapled peptide 4); (E) TPF signal from FITC; (F) overlay of (D) and (E). C≡C image background subtracted using off-resonance image and modified with false colouring for clarity. Scale bars = 25 μm.

The initial spectrum of the diyne stapled peptide 4 was obtained as a solid.^19^ However, to confirm the suitability of diyne stapled peptide 4 for cellular imaging, it was important to assess the Raman intensity of the alkyne resonance of the diyne girder in solution at physiologically relevant concentrations, thus proving the validity of our desired application to employ the diyne staple as a cellular probe.^34^ A solution of the diyne stapled peptide 4 (10 mM in 1 mM PBS) was imaged using a stimulated Raman scattering (SRS) microscope.^35^ In accordance with the solid-state spontaneous Raman spectroscopy experiment, a hyperspectral SRS scan revealed a maximum at 2249 cm^−1^ (Fig. 6(A)). Based on the Raman spectroscopy findings for both the solid-state and solution-phase diyne-girder stapled peptide 4, we thus sought to extend this novel peptide visualisation technique to cellular imaging.

ES-2 ovarian cancer cells were incubated with a 100 μM solution of the Raman active diyne-girder stapled peptide 4 for 3 h, then imaged using confocal SRS microscopy. The localisation of cells was confirmed by imaging at 2940 cm^−1^, corresponding to the CH_3_ vibrational frequency (Fig. 6(B)), highlighting protein distribution throughout the cell and nucleus. Upon tuning the SRS microscope to 2250 cm^−1^, corresponding to the alkyne vibration found in diyne 4 (Fig. 6(C)), a more diffuse signal was observed as expected. Small molecules typically need local intracellular concentrations of low millimolar for successful visualisation by SRS.^36–38^ Pleasingly, this preliminary imaging suggests successful entry of the diyne stapled peptide 4 into cells without the requirement of significant local concentration in specific cellular organelles.

Employing the two-photon fluorescence (TPF) capability of our multiphoton microscope in combination with SRS imaging,^35^ highlighted the instrument's ability to provide cellular localisation without additional staining. HeLa cells were incubated with a 10 μM solution of 4FITC. Cellular location was achieved through SRS imaging of the CH vibration (Fig. 6(D)). Through direct overlay of the fluorescent signal from 4FITC obtained during TPF imaging (Fig. 6(E)) we herein provide images confirming the intracellular localisation of 4FITC (Fig. 6(F)). Line plots of signal intensity across cells identified by their CH_3_ stretching vibrations (Fig. S39, ESI†), highlight cellular features such as nucleoli (with notably higher local protein concentrations) and clearly show the correlation between TPF and CH_3_ signals inside cells.

Herein we have demonstrated SRS cellular imaging of a diyne-girder stapled peptide 4 for the first time. We believe this innovative approach to visualising bioactive peptides within cellular environments, without relying on additional fluorophores, marks a significant advancement in chemical biology by facilitating real-time, non-invasive tracking of intracellular peptides.

## Conclusions

Peptide stapling is a powerful strategy to conformationally constrain α-helical peptides into their bioactive conformation and to improve their overall physiochemical properties. In this work we applied our recently developed diyne-girder α-helical peptide stapling chemistry to the MDM2/MDMX binding domain of p53. By incorporating the necessary unnatural amino acid to achieve the optimal *i*, *i* + 7 bridge length, we developed an α-helical stapled peptide, 4, as assessed by circular dichroism. This peptide showed enhanced helical structure compared to the previously reported alkene-stapled peptides (2/3). Additionally, our diyne-girder stapled peptide 4 demonstrated low nM binding affinity for MDM2, as evaluated by fluorescence polarisation. Notably, this peptide showed a distinctive selectivity for MDM2 over MDMX, with a substantial reduction (∼100-fold) in binding affinity to MDMX. Peptide 4, with pronounced selectivity for MDM2, represents a promising tool compound to interrogate MDM2 cellular activity. We investigated the origin of this selectivity through molecular modelling and docking studies, which provided insight into the specific binding of our diyne-girder stapled peptide 4 to MDM2. Our analysis identified a critical residue in the MDMX binding site that appears to significantly diminish the hydrophobic interactions of peptide 4 that are crucial for binding. Although diyne-girder stapling enhanced the helicity of the peptide, it also decreased the torsional flexibility of the staple when compared to the alkene hydrocarbon constraint, leading to fewer overall hydrophobic interactions with MDMX. This reduction in key interactions accounts for the lower binding affinity observed in fluorescence polarisation experiments. The final aspect of our work concentrated on visualising the diyne-girder stapled peptide within a cellular environment. The unique diyne-girder conformational constraint enabled detection of the peptide by Raman spectroscopy in the “cell silent” region, providing an opportunity for biological visualisation of stapled peptides without the need for the addition of large hydrophobic fluorophores. Notably, among our diyne and alkene analogues, only the diyne-girder analogue exhibited this distinct feature.

## Author contributions

D. C. M. synthesised the compounds, modelled and docked the compounds, and ran all the experiments unless stated otherwise. L. M. and A. K. were involved in the optimisation and synthesis of the amino acid. C. F. S. performed the solution and cellular Raman experiments and the fluorescence cell permeability assay. D. T. H., A. N. H. and A. G. J. provided supervision and funding acquisition. D. C. M., O. A. S. and A. G. J. wrote the manuscript. All authors read and approved the manuscript.

## Data availability

The data supporting this article have been included as part of the ESI.†

## Conflicts of interest

A patent application [(GB) patent application no: 2219576.2] has been submitted on this work. D. T. H. is a consultant for Triana Biomedicines. There are no other conflicts to declare.

## Supplementary Material

CB-OLF-D4CB00288A-s001
