# Supplementary material for: Raman active diyne-girder conformationally constrained p53 stapled peptides bind to MDM2 for visualisation without fluorophores
Source: RSC Chem Biol. 2025 Jan 15;6(3):394–403. doi: 10.1039/d4cb00288a (PMC11741006; doi:10.1039/d4cb00288a)
Supplement: CB-006-D4CB00288A-s001 [file CB-006-D4CB00288A-s001.pdf]

Supplementary Information (SI) for RSC Chemical Biology.
This journal is © The Royal Society of Chemistry 2025

Supporting Information

Raman Active Diyne-Girder Conformationally Constrained p53 Stapled Peptides Bind to MDM2 for Visualisation Without Fluorophores

Danielle C. Morgan[a], Laura McDougall[a], Astrid Knuhtsen[a], Lori Buetow[b], Craig F. Steven[c], Oscar A. Shepperson[a], Danny T. Huang[b,d], Alison N. Hulme[c] and Andrew G. Jamieson[a,†].

[a] *School of Chemistry, Advanced Research Centre, University of Glasgow, 11 Chapel Lane, Glasgow, G11 6EW, UK.*
[b] *Cancer Research UK Scotland Institute, Garscube Estate, Switchback Road, Glasgow, G61 1BD, UK.*
[c] *EaStCHEM School of Chemistry, The University of Edinburgh, West Mains Road, Edinburgh, EH9 3JJ, UK.*
[d] *School of Cancer Sciences, University of Glasgow, Glasgow, G61 1QH, UK.*
†Corresponding Author: Andrew G. Jamieson - E-mail: andrew.jamieson.2@glasgow.ac.uk

Table of Contents

Experimental Procedures 3

General Information 3

Peptide Synthesis - General Methods 4
- General Method 1: Peptide Synthesis 4
- General Method 2: N-Terminal Acetylation 4
- General Method 3: Coupling of Fluorescein Isothiocyanate (FITC) 4
- General Method 4: Ring Closing Metathesis (RCM) 4
- General Method 5: Glaser Reaction 4
- General Method 6: Peptide Cleavage and Global Deprotection 5
- General Method 7: Peptide Purification 5

Protocol for Synthesis of Peptides 1 – 5 and 1FITC – 5FITC 6
- Peptide 1 6
- Peptide 1FITC 7
- Peptide 2/3 8
- Peptide 2FITC/3FITC 9
- Peptide 4 10
- Peptide 4FITC 11
- Peptide 5 12
- Peptide 5FITC 13

Circular Dichroism (CD) Spectroscopy 14

Cell Permeability Fluorescence Assay 14

Cellular Raman Spectroscopy 15

Fluorescence (two-photon) Imaging 15

Fluorescence + SRS (multiphoton) Imaging 15

Solid-state Raman Spectroscopy of Native and Diyne Stapled Peptides 16

Production of MDM2/MDMX Proteins 16

Fluorescence Polarisation Binding Assay 17

Synthesis of Unnatural Amino Acids 18

Peptide Characterisation Data 19
- Peptide Summary Characterisation Table 19
- Spectral Data: RP-HPLC 21
- Spectral Data: ESI-MS 31

Supporting Information Tables and Figure 34

References 43

Experimental Procedures

General Information

All reagents were purchased from commercial sources and used without further purification unless otherwise stated. Fmoc-protected amino acids were purchased from CEM Corporation, and Pepceuticals. *N,N*-Dimethylformamide (DMF) and diethyl ether (Et_2O) were purchased from Rathburn. (*R*)-*N*-Fmoc-*α*-(7-octenyl)alanine (R_8), (*S*)-*N*-Fmoc-*α*-(4-pentenyl)alanine (S_5), triisopropylsilane (TIPS), 1,2-dichloroethane (DCE) and Grubbs 1st Catalyst were purchased from Sigma Aldrich. Trifluoroacetic acid (TFA), *N,N*-diisopropylethylamine (DIPEA), *N,N*-diisopropylcarbodiimide (DIC), ethyl (hydroxyamino)cyanoacetate (Oxyma Pure), fluorescein-5-isothiocyanate (FITC) and Fmoc-6-Ahx-OH, were purchased from Fluorochem. Morpholine was purchased from Alfa Aesar. Dichloromethane (CH_2Cl_2) was purchased from VWR. Acetonitrile (MeCN) was purchased from Fisher Scientific. ChemMatrix© Rink-Amide resin was purchased from Biotage. All other reagents were purchased from Sigma Aldrich. Dry solvents were purified using a PureSolv 500 MD solvent purification system.

Analytical reverse-phase high-performance liquid chromatography (RP-HPLC) was performed on a Shimadzu RP-HPLC system with Shimadzu LC-20AT pumps, a Shimadzu SIL20A autosampler and a Shimadzu SPD-20A UV-vis detector using a Phenomenex Aeris C18 (100Å, 5 µm, 150 × 4.6 mm). Compounds were eluted with linear gradients at column-dependent flow rates (1 mL/min for the Aeris), where buffer A = 0.1% TFA in H_2O and buffer B = 0.1% TFA in MeCN. Data is reported as column retention time (t_R) in minutes (min). Crude peptides were purified by preparative RP-HPLC using a Dionex RP-HPLC system with Dionex P680 pumps and a Dionex UVD170U UV-Vis detector (monitoring at 214 nm and 280 nm), with a Phenomenex Gemini column 5 µm C18 column (100 Å, 250 x 21.2 mm). Peptides were eluted on linear gradients (10 mL/min) as determined by analytical RP-HPLC. The solvents employed were buffer; A = H_2O + 0.1% TFA, and buffer; B = MeCN + 0.1% TFA.

Liquid chromatography-mass spectrometry (LC-MS) was performed on a Thermo Scientific LCQ Fleet Ion Trap Mass Spectrometer using positive mode electrospray ionisation (ESI^+). Where buffer A = 0.1 % TFA in 95% H_2O/5% MeCN and buffer B = 0.1% TFA in 95% MeCN/5% H_2O, a linear gradient of 5-95% B over 20 min with a flow rate of 1 mL/min was used with a Reprosil-Gold column (3 mm C18, 150 x 4 mm).

Microwave reactions were completed in a CEM Explorer 12 Hybrid Microwave. Peptide content was analysed on a Nanodrop 2000c using UV absorption of peptides at 280 nm or 214 nm. Samples were centrifuged at 4,500 RPM in a Heraeus Megafuge 8 centrifuge purchased from Thermo Fisher Scientific.

Peptide Synthesis - General Methods

General Method 1: Peptide Synthesis

Peptides were synthesised on an Automated Biotage Initiator+ Alstra microwave synthesiser on a 0.1 mmol scale. The peptides were synthesised using Rink-Amide ChemMatrix© resin (0.49 mmol/g, 0.204 mg). Peptides were elongated in cycles of amino acid coupling followed by Fmoc removal. Fmoc-protected amino acid (5 equiv., 0.2 M in DMF) and unnatural/orthogonally protected amino acid (2 equiv., 0.1 M in DMF) coupling was achieved by treatment with DIC (5 equiv., 0.5 M in DMF) and Oxyma Pure (5 equiv., 0.5 M in DMF) at 90 °C for 2 min respectively. Amino acids following unnatural amino acids were double coupled. Fmoc removal was achieved by treatment with 20% morpholine and 5% formic acid (*v/v*) in DMF (4 mL) for 90 °C for 1 min. The resin was washed with DMF following Fmoc removals (4 x 4 mL), and after coupling (2 x 4 mL).

General Method 2: N-Terminal Acetylation

Peptides requiring N-terminal acetylation were treated on-resin with acetic anhydride (3 equiv.), DIPEA (5 equiv.) and DMF (7 mL for 0.1 mmol of resin) for 20 min with agitation. The resin was then washed with DMF (3 x 5 mL) and CH_2Cl_2 (3 x 5 mL) prior to peptide cleave and global deprotection.

General Method 3: Coupling of Fluorescein Isothiocyanate (FITC)

Peptides requiring an N-terminal fluorescent label were treated on-resin with FITC (2 equiv.), DIPEA (8 equiv.) and DMF (4 mL for 0.1 mmol of resin) at room temperature and excluding light for 16 h. The resin was then washed with DMF (2 x 5 mL) and CH_2Cl_2 (2 x 5 mL) prior to peptide cleave and global deprotection.

General Method 4: Ring Closing Metathesis (RCM)

Peptides containing (*R*)-*N*-Fmoc-*α*-(7-octenyl)alanine and (*S*)-*N*-Fmoc-*α*-(4-pentenyl)alanine were stapled by on-resin ring-closing metathesis (RCM). The resin was suspended in dry DCE before adding Grubbs 1st Catalyst (20 mol%) in DCE (4 mL for 0.1 mmol of resin), leaving for 2 h with agitation and excluding light. The resin was washed with DCE (3 mL) before repeating the RCM. The resin was then washed with CH_2Cl_2 (2 x 5 mL) prior to peptide cleave and global deprotection.

General Method 5: Glaser Reaction

Peptides containing (S)-2-((((9H-Fluoren-9-yl)methoxy)carbonyl)amino)-2-methylnon-8-ynoic acid were cyclised on resin. Peptide on resin (1 equiv., 0.1 mmol) was added to a

microwave vial followed by a solution of CuCl (5 equiv.) and 4,4'-bis(hydroxymethyl)-2,2'-bipyridine (7.5 equiv.) in DMF (10 mL) and DIPEA (20 equiv.). The reaction was heated to 60 °C for 3 h in a microwave reactor and excess reagents filtered off. The resin was washed with 0.5% sodium diethyldithiocarbamate in 0.5% DIPEA/DMF (5 x 10 mL), DMF (3 x 10 mL), methanol (MeOH) (3 x 10 mL), DMF (3 x 10 mL) and CH_2Cl_2 (3 x 10 mL).

General Method 6: Peptide Cleavage and Global Deprotection

Peptides were cleaved from the resin using a cocktail (10 mL) of TFA (95%), TIPS (2.5%) and H_2O (2.5%) for 4 h with agitation. The resin was subsequently filtered and the TFA evaporated using a stream of N_2. The peptide was precipitated with cold Et_2O and centrifuged (4,500 rpm for 5 min). Peptides were dissolved in a 1:1 mixture (*v/v*) H_2O and MeCN with 0.1% TFA and lyophilised.

General Method 7: Peptide Purification

Crude peptides were purified by preparative RP-HPLC using a Dionex RP-HPLC system with Dionex P680 pumps and a Dionex UVD170U UV-Vis detector (monitoring at 214 nm and 280 nm), with a Phenomenex Gemini column 5 µm C18 column (100 Å, 250 x 21.2 mm). Peptides were eluted on linear gradients (10 mL/min) as determined by analytical RP-HPLC. The solvents employed were buffer; A = H_2O + 0.1% TFA, and buffer; B = MeCN + 0.1% TFA.

Protocol for Synthesis of Peptides 1 – 5 and 1FITC – 5FITC

Peptide 1

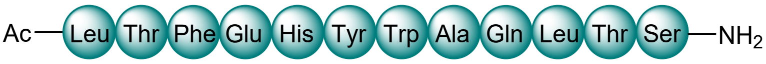

Peptide **1** was elongated by automated Fmoc-SPPS as outlined by **General Method 1**. Following complete elongation of the on-resin peptide, the peptidyl resin was N-terminally capped as outlined by **General Method 2**. The final linear peptide was liberated from the resin and protecting groups simultaneously removed following **General Method 6**, to afford the crude linear peptide (**1**) as a white amorphous powder. The crude peptide was purified as described by **General Method 7** and lyophilised to yield the desired final peptide as a white amorphous powder, 23% yield, > 99% purity.

ESI-MS: Mass calculated for [$C_{73}H_{101}N_{17}O_{20}$] 1535.74; deconvoluted mass observed: 1536.00 ± 0.23. Charge states; 769.08 $[M+2H]^{2+}$, 1536.83 $[M+H]^{+}$.

RP-HPLC: t_R = 14.8 min. Phenomenex Aeris C18 (100Å, 5 μm, 150 × 4.6 mm). Linear gradient 5%B – 95%B over 20 mins (*ca.* 4.5%B/min) at 1 mL/min.

Peptide 1FITC

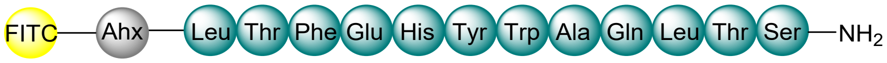

Peptide **1FITC** was elongated by automated Fmoc-SPPS as outlined by **General Method 1**. Following complete elongation of the on-resin peptide, the peptidyl resin was N-terminally tagged as outlined by **General Method 3**. The final linear peptide was liberated from the resin and protecting groups simultaneously removed following **General Method 6**, to afford the crude linear peptide (**1FITC**) as a yellow amorphous powder. The crude peptide was purified as described by **General Method 7** and lyophilised to yield the desired final peptide as a yellow amorphous powder, 22% yield, > 99% purity.

ESI-MS: Mass calculated for [$C_{98}H_{120}N_{19}O_{25}S$] 1996.85; deconvoluted mass observed: 1996.13. Charge states; 999.25 $[M+2H]^{2+}$.

RP-HPLC: t_R = 15.7 min. Phenomenex Aeris C18 (100Å, 5 µm, 150 × 4.6 mm). Linear gradient 5%B – 95%B over 20 mins (*ca.* 4.5%B/min) at 1 mL/min.

Peptide 2/3

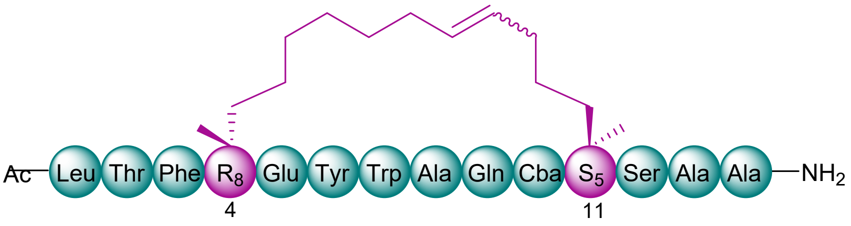

Peptides **2/3** were elongated by automated Fmoc-SPPS as outlined by **General Method 1**. Following complete elongation of the on-resin peptide, the peptidyl resin was N-terminally capped as outlined by **General Method 2**. The linear peptidyl resin was stapled in accordance with **General Method 4** to yield a mixture of *cis-/trans-* isomers. The mixture of stapled peptides was liberated from the resin and protecting groups simultaneously removed following **General Method 6**, to afford the crude stapled peptides (**2/3**) as white amorphous powders. The crude peptides were purified as described by **General Method 7** and collected as pure isomers and lyophilised to yield the desired final peptides as white amorphous powders, 4%/2% yield, > 99%/> 97% purity.

ESI-MS: Mass calculated for [$C_{87}H_{125}N_{17}O_{21}$] 1743.92; deconvoluted mass observed: (**2**) 1743.87 ± 0.41. Charge states; 873.08 $[M+2H]^{2+}$, 1744.58 $[M+H]^{+}$. (**3**) 1744.01 ± 0.47. Charge states; 873.17 $[M+2H]^{2+}$, 1744.67 $[M+H]^{+}$.

RP-HPLC: t_R = (**2/3**) 21.6/22.4 min. Phenomenex Aeris C18 (100Å, 5 µm, 150 × 4.6 mm). Linear gradient 5%B – 95%B over 20 mins (*ca.* 4.5%B/min) at 1 mL/min.

Peptide 2FITC/3FITC

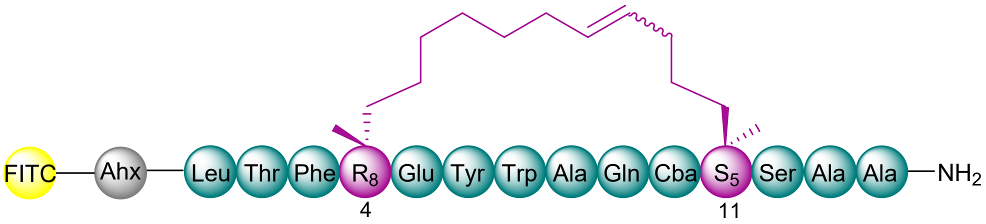

Peptides **2FITC/3FITC** were elongated by automated Fmoc-SPPS as outlined by **General Method 1**. Following complete elongation of the on-resin peptide, the peptidyl resin was N-terminally tagged as outlined by **General Method 3**. The linear peptidyl resin was stapled in accordance with **General Method 4** to yield a mixture of *cis-/trans-* isomers. The mixture of stapled peptides was liberated from the resin and protecting groups simultaneously removed following **General Method 6**, to afford the crude stapled peptides (**2FITC/3FITC**) as yellow amorphous powders. The crude peptides were purified as described by **General Method 7** and collected as pure isomers and lyophilised to yield the desired final peptides as yellow amorphous powders, 4%/2% yield, > 99%/> 97% purity.

ESI-MS: Mass calculated for [$C_{112}H_{145}N_{19}O_{26}S$] 2205.04; deconvoluted mass observed: (**2FITC**) 2204.34. Charge states; 1103.17 $[M+2H]^{2+}$. (**3FITC**) 2204.34. Charge states; 1103.17 $[M+2H]^{2+}$.

RP-HPLC: (**2FITC/3FITC**) t_R = 21.8/22.1 min. Phenomenex Aeris C18 (100Å, 5 µm, 150 × 4.6 mm). Linear gradient 5%B – 95%B over 20 mins (*ca.* 4.5%B/min) at 1 mL/min.

Peptide 4

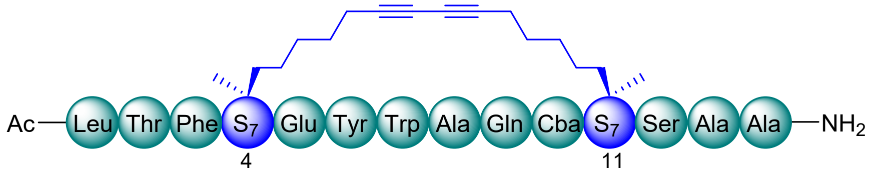

Peptide **4** was elongated by automated Fmoc-SPPS as outlined by **General Method 1**. Following complete elongation of the on-resin peptide, the peptidyl resin was N-terminally capped as outlined by **General Method 2**. The linear peptidyl resin was stapled in accordance with **General Method 5**. The stapled peptide was liberated from the resin and protecting groups simultaneously removed following **General Method 6**, to afford the crude stapled peptide (**4**) as a white amorphous powder. The crude peptide was purified as described by **General Method 7** and lyophilised to yield the desired final peptide as a white amorphous powder, 10% yield, > 97% purity.

ESI-MS: Mass calculated for [$C_{90}H_{125}N_{17}O_{21}$] 1779.92; deconvoluted mass observed: 1780.05 ± 0.42. Charge states; 891.17 $[M+2H]^{2+}$, 1780.75 $[M+H]^{+}$.

RP-HPLC: t_R = 21.4 min. Phenomenex Aeris C18 (100Å, 5 µm, 150 × 4.6 mm). Linear gradient 5%B – 95%B over 20 mins (*ca.* 4.5%B/min) at 1 mL/min.

Peptide 4FITC

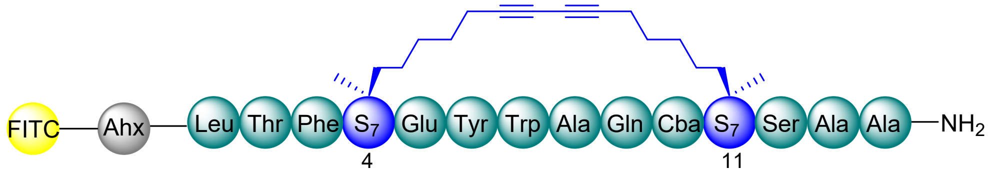

Peptide **4FITC** was elongated by automated Fmoc-SPPS as outlined by **General Method 1**. Following complete elongation of the on-resin peptide, the peptidyl resin was N-terminally tagged as outlined by **General Method 3**. The linear peptidyl resin was stapled in accordance with **General Method 5**. The stapled peptide was liberated from the resin and protecting groups simultaneously removed following **General Method 6**, to afford the crude stapled peptide (**4FITC**) as a yellow amorphous powder. The crude peptide was purified as described by **General Method 7** and lyophilised to yield the desired final peptide as a yellow amorphous powder, 9% yield, > 97% purity.

ESI-MS: Mass calculated for [$C_{115}H_{145}N_{19}O_{26}S$] 2240.04; deconvoluted mass observed: 2239.84. Charge states; 1120.92 $[M+2H]^{2+}$.

RP-HPLC: t_R = 21.5 min. Phenomenex Aeris C18 (100Å, 5 µm, 150 × 4.6 mm). Linear gradient 5%B – 95%B over 20 mins (*ca.* 4.5%B/min) at 1 mL/min.

Peptide 5

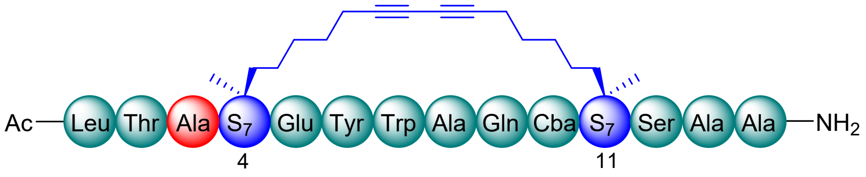

Peptide **5** was elongated by automated Fmoc-SPPS as outlined by **General Method 1**. Following complete elongation of the on-resin peptide, the peptidyl resin was N-terminally capped as outlined by **General Method 2**. The linear peptidyl resin was stapled in accordance with **General Method 5**. The stapled peptide was liberated from the resin and protecting groups simultaneously removed following **General Method 6**, to afford the crude stapled peptide (**5**) as a white amorphous powder. The crude peptide was purified as described by **General Method 7** and lyophilised to yield the desired final peptide as a white amorphous powder, 11% yield, > 99% purity.

ESI-MS: Mass calculated for [$C_{84}H_{121}N_{17}O_{21}$] 1703.89; deconvoluted mass observed: 1703.54 ± 0.17. Charge states; 852.83 $[M+2H]^{2+}$, 1704.42 $[M+H]^{+}$.

RP-HPLC: t_R = 19.6 min. Phenomenex Aeris C18 (100Å, 5 µm, 150 × 4.6 mm). Linear gradient 5%B – 95%B over 20 mins (*ca.* 4.5%B/min) at 1 mL/min.

Peptide 5FITC

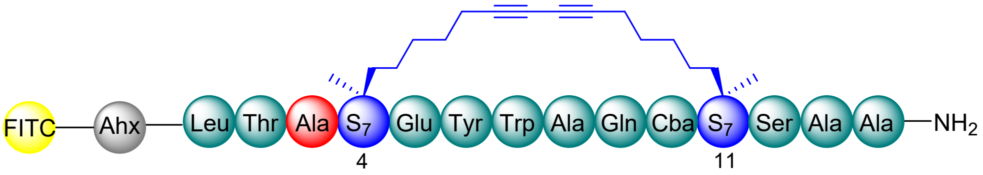

Peptide **5FITC** was elongated by automated Fmoc-SPPS as outlined by **General Method 1**. Following complete elongation of the on-resin peptide, the peptidyl resin was N-terminally tagged as outlined by **General Method 3**. The linear peptidyl resin was stapled in accordance with **General Method 5**. The stapled peptide was liberated from the resin and protecting groups simultaneously removed following **General Method 6**, to afford the crude stapled peptide (**5**) as a yellow amorphous powder. The crude peptide was purified as described by **General Method 7** and lyophilised to yield the desired final peptide as a yellow amorphous powder, 9% yield, > 98% purity.

ESI-MS: Mass calculated for [$C_{109}H_{140}N_{19}O_{26}S$] 2164.00; deconvoluted mass observed: 2163.84. Charge states; 1082.92 $[M+2H]^{2+}$.

RP-HPLC: t_R = 20.5 min. Phenomenex Aeris C18 (100Å, 5 µm, 150 × 4.6 mm). Linear gradient 5%B – 95%B over 20 mins (*ca*. 4.5%B/min) at 1 mL/min.

Circular Dichroism (CD) Spectroscopy

CD spectra were obtained at room temperature using a JASCOJ-810 CD spectrometer. A range of 190-260 nm was scanned at a speed of 50 nm/min, with a 1 nm data pitch, a 1 nm bandwidth, and an 8 s response time. Samples were prepared (50 μM) in phosphate buffered saline (PBS; pH 7.4), and CD spectra measured in a 1 mm or 0.2 mm quartz cuvette. Raw data (mdeg) were converted to mean residue ellipticity (MRE; deg cm^2 $dmol^{-1}$ res^{-1}) by normalising for path length, peptide concentration, and number of amide bonds. Percentage helicities can be calculated for α-helical peptides. The raw CD data was converted to mean residue ellipticity (MRE) using Equation S1, and the value at 222 nm was used to calculate the % helicity of the peptides using Equation S2.[1,2]

Equation S1. MRE calculation. Where θ = machine units in degrees, MRW (mean residue weight) = molecular mass of peptide/number of residues, l = path length (cm) and c = peptide concentration in mg/mL.

$$MRE = \theta \left(\frac{0.1 \times MRW}{l \times c} \right)$$

Equation S2. (**1**) % Helicity equation. (**2**) θ_c = random coil ellipticity calculation proposed by Luo and Baldwin.[1] (**3**) $\theta_{222\infty}$ = α-helix ellipticity calculation determined by Luo and Baldwin,[3] reading observed at 222 nm. T is the temperature in degrees Celsius, Np is the number of amide bonds and *k* = the peptide length correction factor, 3.[2]

$$(1)\ \%\, Helicity = \left(\frac{\theta_{222} - \theta_c}{\theta_{222\infty} - \theta_c} \right) \times 100$$

$$(2)\ \theta_c = 2220 - 53T$$

$$(3)\ \theta_{222\infty} = (-44000 + (250 \times T)) \times \left(1 - \frac{k}{Np}\right)$$

Cell Permeability Fluorescence Assay

HEK293 cells were cultured in Dulbecco's Modified Eagle Medium (DMEM, high glucose with GlutaMAX, Gibco) supplemented with 10% (v/v) foetal bovine serum (Gibco) and 1% (v/v) penicillin/streptomycin (10,000 units/mL penicillin, 10,000 μg/mL streptomycin, Gibco). Cultured cells were maintained in a humidified incubator at 37 °C, 5% CO_2 and passaged twice weekly in T-25 flasks (Corning). For cell counting, an aliquot (10 μL) of cell solution in media was added to a haemocytometer slide which was viewed using a microscope for manual inspection and counting. For experiments, 300,000 cells were seeded into 6-well plates (CytoOne) on 30 mm cover glass slides pre-treated with 0.1 mg/mL poly-D-lysine and left to grow for two days to reach ca. 80% confluency before compound incubation. Media was removed and the cells were washed with PBS prior to treatment with compound in PBS (2 h,

20 μM, 37 °C). Cells were then washed again with PBS twice, fixed with a 4% (w/v) solution of formaldehyde in PBS (10 min, 37 °C) and washed with PBS twice prior to analysis.

Fluorescence Imaging

Images were acquired on a MetaMorph/Metafluor fluorescence imaging microscope system equipped with a 40× Superfluor objective for an exposure time of 1000 ms. Excitation for fluorescein was conducted at 495 nm. Image analysis and processing was performed using MetaMorph microscopy software.

Cellular Raman Spectroscopy

ES-2 (human ovarian cancer) and HeLa (human cervical cancer) cells were used. Cell lines were cultured in Dulbecco's Modified Eagle Medium (DMEM, high glucose, Gibco), supplemented with 10% (*v/v*) foetal bovine serum (Gibco), 1% (*v/v*) L-glutamine (200 mM, Gibco), and 1% (*v/v*) penicillin/streptomycin (10,000 units/mL penicillin, 10,000 μg/mL streptomycin, Gibco). Cultured cells were maintained in a humidified incubator at 37 °C and 5% CO_2. Cells were plated in FluoroDish Cell Culture Dishes (35 mm, World Precision Instruments) in DMEM (phenol red-free, Gibco) and allowed to reach *ca*. 80% confluency overnight before compound incubation. Cells were treated with compounds in DMEM (3 h, 20 – 100 μM, 37 °C), washed with Dulbecco's phosphate buffered saline (PBS) twice, fixed with a 4% (*w/v*) solution of formaldehyde in PBS (10 min, 37 °C), washed with PBS twice, incubated with Hoechst 33342 in PBS (1 μM, 15 min, 37 °C) and finally washed with PBS twice.

Fluorescence (two-photon) Imaging

Images were acquired on an Olympus FV3000 laser scanning confocal microscope equipped with a 40× or 60× objective. Image analysis and processing was performed using ImageJ 1.53c.

Fluorescence + SRS (multiphoton) Imaging

Images were acquired using a custom-built multi-modal microscope setup, as previously described in detail.[3] Briefly, a picoEmerald S (APE, Berlin, Germany) laser provided both a tunable pump laser (700–990 nm, 2 ps, 80 MHz repetition rate) and a spatially and temporally overlapped Stokes laser (1032nm, 2 ps, 80 MHz repetition rate). The output beams were inserted into the scanning unit of an Olympus FV1000MPE microscope using a series of dielectric mirrors and a 3× lens-based beam-expanding module. The resulting 3.6 mm beams were expanded by a further 3.6× lens within the microscope and directed into an Olympus XLPL25XWMP N.A. 1.05 objective lens using a short-pass 690 nm dichroic mirror (Olympus). For SRS measurements the Stokes beam was intensity modulated with a 20 MHz EoM built into the picoEmerald S. Forward scattered light was collected by a further 25× Olympus XLPL25XWMP N.A. 1.05 objective lens and Stokes light was removed by filtering

with an ET890/220m filter (Chroma). A telescope focused the light onto an APE silicon photodiode connected to an APE lock in amplifier with the time constant set to 20 µs. The lock in amplifier signal was fed into an Olympus FV10-Analog unit. Laser powers after the objective were measured up to 20–50 mW for the pump laser and up to 70 mW for the Stokes laser. All images were recorded at 512 × 512 pixels (hyperspectral scans) or 1024 × 1024 pixels (normal imaging) with a pixel dwell time between 2 and 20 µs, using FluoView FV10-ASW scanning software (Olympus). Image analysis and processing was performed using ImageJ 1.53c. Hyperspectral images (obtained from a drop of concentrated sample in DMSO or PBS) were recorded using the inbuilt 'sweep' function of the picoEmerald S software that adjusted the pump laser ~0.3 nm for each new image recorded. Hyperspectral images were analysed using the 'Measure Stack' feature on ImageJ 1.53c to quantify the change in signal intensity. Off-resonance images were acquired by tuning to a wavelength ca. ± 2 nm and were used to subtract background from alkyne on-resonance images.

Solid-state Raman Spectroscopy of Native and Diyne Stapled Peptides

Raman spectroscopy was performed using the Horiba Jobin Yvon LabRAM HR system with a Ventus CD laser at 532.02 nm, 100 mW. The hole width was 200 µM with a diffraction grating of 600 g/mm using an Olympus x50LWD objective lens. The recorded spectral range was 600-4000 cm^{-1} and data acquisition was performed during 5 seconds with 3 repeats and collected with the Synapse OCD detector. 100% power was used for peptide **40** and 50% power was used for stapled peptide **41**. Data was analysed using the Labspec 5 software.

Production of MDM2/MDMX Proteins

Polymerase chain reaction was used to amplify the regions encoding human MDM2 1-138 and human MDM4 1-134 from synthetic *MDM2* and *MDM4* gene fragments (Integrated DNA Technologies) codon optimised for expression in *Escherichia coli*. The amplified products were cloned by restriction enzyme digest and ligation into pGEX-4T-1 (Cytiva) vector in which the thrombin protease cleavage site was replaced with a tobacco etch virus (TEV) cleavage site and a non-cleavable hexahistidine sequence was inserted prior to the glutathione S-transferase affinity tag (His_6-GST). The MDM2-encoding plasmid was transformed into *E. coli* Rosetta™ 2(DE3)pLac1 (Novagen) cells and the MDM4-encoding plasmid into *E. coli* Rosetta™ 2(DE3)pLysS (Novagen) cells for recombinant protein expression. For MDM2 1-138 production, cells were grown in Luria broth (Miller) at 37 °C until an optical density of 0.7-0.8 was reached; protein expression was then induced with 0.2 mM isopropyl β-D-1-thiogalactopyranoside at 20 °C for 18 hours. For MDM4 1-134 production, cells were grown in autoinduction media at 37 °C for 8 hours and subsequently the temperature was reduced to 20 °C for 16 hours. Cells were harvested by centrifugation at 4500 g and resuspended in Dulbecco's phosphate buffered saline (PBS, Thermo Scientific™ Oxoid™) supplemented with 330 mM NaCl, 40 mM imidazole, and 5 mM beta-mercaptoethanol (BME). Cells were lysed using a microfluidizer and clarified lysate was recovered by centrifuging at 48,000 g for 1 hour at 4 °C. Clarified lysate was applied to a HisTrapFF column (Cytiva) and washed with

resuspension buffer. The His_6-GST-tagged proteins were eluted with buffer comprising PBS, 400 mM imidazole, and 5 mM BME and then applied to a GSTrap FF column (Cytiva) and washed with 200 mM NaCl, 50 mM Tris-HCl, pH 7.6, 1 mM DTT. The proteins were eluted with 100 mM NaCl (His_6-GST-MDM4 1-134) or 200 mM NaCl (His_6-GST-MDM2 1-138), 50 mM Tris-HCl, pH 8.0, 5 mM dithiothreitol (DTT), 10 mM glutathione. His_6-GST-MDM4 1-134 was further purified by anion exchange chromatography using a NaCl gradient at pH 9. The His_6-GST -tagged proteins were then cleaved with TEV while dialyzing into 330 mM NaCl, PBS, 5 mM BME for 20 hours at 4 °C. The cleaved products were applied to a HisTrapFF column to remove TEV protease and the affinity tag, and subsequently further purified on a HiLoad Superdex 75 pg size exclusion column (Cytiva) in 150 mM NaCl, 25 mM Tris-HCl, pH 7.6, 1 mM dithiothreitol. The purified proteins were snap frozen in liquid nitrogen and stored at -80 °C. Protein concentration was measured at an absorbance of 595 nm with the Bio-rad Protein Assay Kit II using bovine serum albumin as a standard.[4]

Fluorescence Polarisation Binding Assay

The binding properties of the peptides were assessed using a biophysical FP assay. Specifically, a direct binding assay was conducted to calculate the K_d values of the fluorescent peptide ligands binding to the target proteins. Fluorescently labelled peptides were tested for binding to MDM2 (1-138) and MDMX (1-134). The proteins were provided by Dr Lori Buetow at the Beatson Cancer Institute in Glasgow. All FP measurements were performed on a microplate reader (CLARIOstar) in black 384-well microplates with 20 µL of sample solution per well. Protein (MDM2 1-138 or MDMX 1-134) in 150 mM NaCl, 25 mM Tris, pH 7.6, 1 mM DTT was added and serially diluted from 10 µM to 9 pM. Peptide (10 nM in Tris buffer pH 7.6) was then added to all wells and mixed. The sample were left to incubate at room temperature for 3 hours. Plates were placed in a PerkinElmer Victor X5 plate reader with excitation measured at 531 nm and emission measured at 595 nm. The raw data was normalized using baseline correction, where the baseline was defined as the fluorescence polarisation measured in the absence of peptide. Normalised data were then plotted in Prism 7.0 and fitted by using the quadratic ligand depletion model (**Equation S3**).

Equation S3. Y is the fluorescent polarisation signal measured at each datapoint. R_t is the total concentration of receptor (FAM-labelled DNA). X is the total concentration of ligand (peptide) and K_d is the dissociation constant (the inflection point of the curve on a logarithmic scale).

$$Y = \frac{K_d + R_t + X - \sqrt{({K_d}^2 + (R_t - X)^2 + 2K_d(R_t + X))}}{2}$$

Synthesis of Unnatural Amino Acids

(*S*)-2-((((9*H*-Fluoren-9-yl)methoxy)carbonyl)amino)-2-methylnon-8-ynoic acid (S_7)

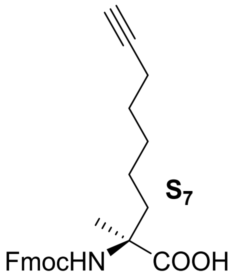

S_7

This unnatural amino acid was synthesised as described by the procedure by Morgan *et al.* (2023).[5]

Peptide Characterisation Data

Peptide Summary Characterisation Table

Table S1. Name, sequence, % yield, % purity, *m/z* and retention time of peptides. Abbreviations: FITC = fluorescein-5-isothiocyanate, Ahx = Aminohexanoic acid, NH_2 = C-terminal amide, Ac = acetylated, Cba = L-Cyclobutylalanine, R_8 = (*R*)-*N*-Fmoc-α-(7-octenyl)alanine, S_5 = (*S*)-*N*-Fmoc-α-(4-pentenyl)alanine, S_7 = (*S*)-2-((((9*H*-Fluoren-9-yl)methoxy)carbonyl)amino)-2-methylnon-8-ynoic acid.

Peptide	Sequence	Yield (%)	% Purity	Calculated *m/z*	Observed *m/z*	t_R 20 min (min)	t_R 50 min (min)
**1**	*Ac-LTFEHYWAQLTS-NH_2*	23	> 99	$[M+2H]^{2+}$ = 796.08	$[M+2H]^{2+}$ = 769.08	14.8	28.1
**1FITC**	*FITC-Ahx-LTFEHYWAQLTS-NH_2*	22	> 99	$[M+2H]^{2+}$ = 998.93	$[M+2H]^{2+}$ = 999.25	15.7	30.5
**2**	*Ac-LTFR_8EYWAQ(Cba)S_5SAA-NH_2*	4	> 99	$[M+2H]^{2+}$ = 872.97	$[M+2H]^{2+}$ = 873.08	21.6	42.2
**2FITC**	*FITC-Ahx-LTFR_8EYWAQ(Cba)S_5SAA-NH_2*	4	> 99	$[M+2H]^{2+}$ = 1103.02	$[M+2H]^{2+}$ = 1103.17	22.4	43.8
**3**	*Ac-LTFR_8EYWAQ(Cba)S_5SAA-NH_2*	2	> 97	$[M+2H]^{2+}$ = 872.97	$[M+2H]^{2+}$ = 873.17	21.8	43.5
**3FITC**	*FITC-Ahx-LTFR_8EYWAQ(Cba)S_5SAA-NH_2*	2	> 97	$[M+2H]^{2+}$ = 1103.02	$[M+2H]^{2+}$ = 1103.17	22.1	44.1

**4**	*Ac-LTFS$_7$EYWAQ(Cba)S$_7$SAA-NH$_2$*	10	> 97	$[M+2H]^{2+}$ = 890.97	$[M+2H]^{2+}$ = 891.17	21.4	41.9
**4FITC**	*FITC-Ahx-LTFS$_7$EYWAQ(Cba)S$_7$SAA-NH$_2$*	9	> 97	$[M+2H]^{2+}$ = 1121.02	$[M+2H]^{2+}$ = 1120.92	21.5	43.1
**5**	*Ac-LTAS$_7$EYWAQ(Cba)S$_7$SAA-NH$_2$*	11	> 99	$[M+2H]^{2+}$ = 851.95	$[M+2H]^{2+}$ = 852.83	19.6	38.3
**5FITC**	*FITC-Ahx-LTAS$_7$EYWAQ(Cba)S$_7$SAA-NH$_2$*	9	> 98	$[M+2H]^{2+}$ = 1082.01	$[M+2H]^{2+}$ = 1082.92	20.5	40.9

Spectral Data: RP-HPLC

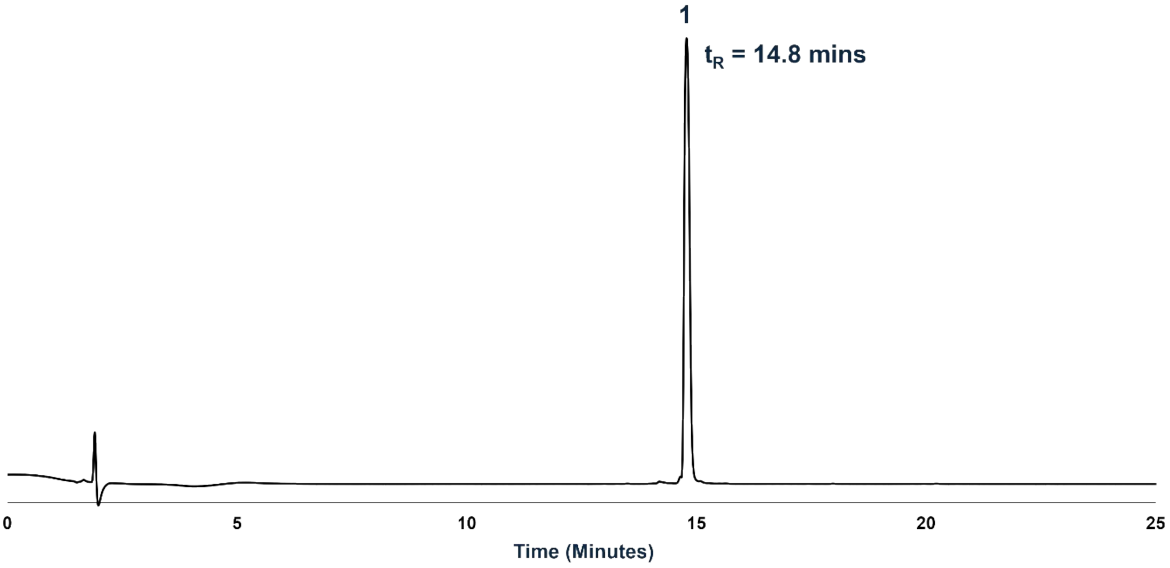

Figure S1. Analytical HPLC chromatogram (214 nm) of purified peptide, **1** (*ca.* > 99% as analysed by peak area. Phenomenex Aeris 5 µm C18 (100Å, 5 µm, 150 × 4.6 mm). Linear gradient 5%B – 95%B over 20 mins (*ca.* 4.5%B/min) at 1 mL/min. t_R = 14.8 mins.

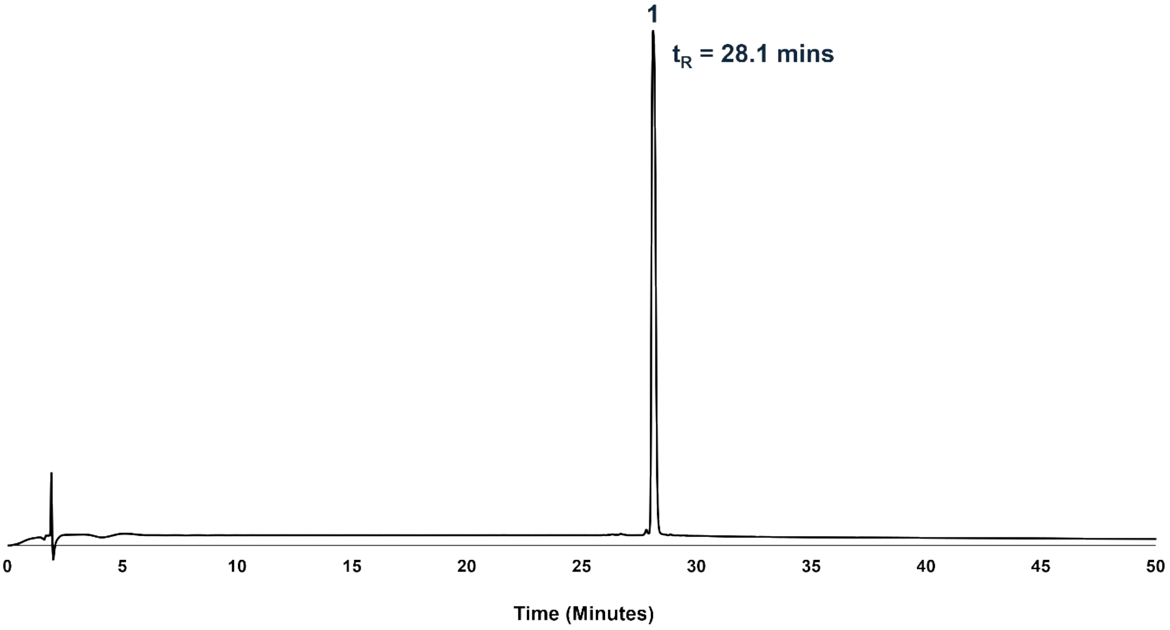

Figure S2. Analytical HPLC chromatogram (214 nm) of purified peptide, **1** (*ca.* > 99% as analysed by peak area. Phenomenex Aeris 5 µm C18 (100Å, 5 µm, 150 × 4.6 mm). Linear gradient 5%B – 95%B over 50 mins (*ca.* 1.8%B/min) at 1 mL/min. t_R = 28.1 mins.

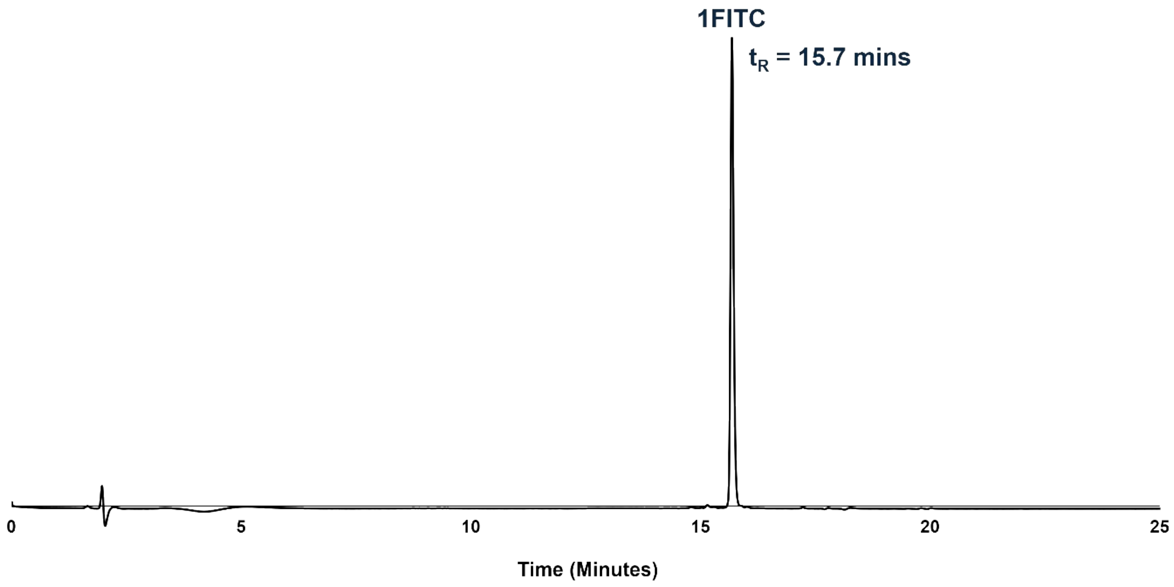

Figure S3. Analytical HPLC chromatogram (214 nm) of purified peptide, **1FITC** (*ca.* > 99% as analysed by peak area. Phenomenex Aeris 5 µm C18 (100Å, 5 µm, 150 × 4.6 mm). Linear gradient 5%B – 95%B over 20 mins (*ca.* 4.5%B/min) at 1 mL/min. t_R = 15.7 mins.

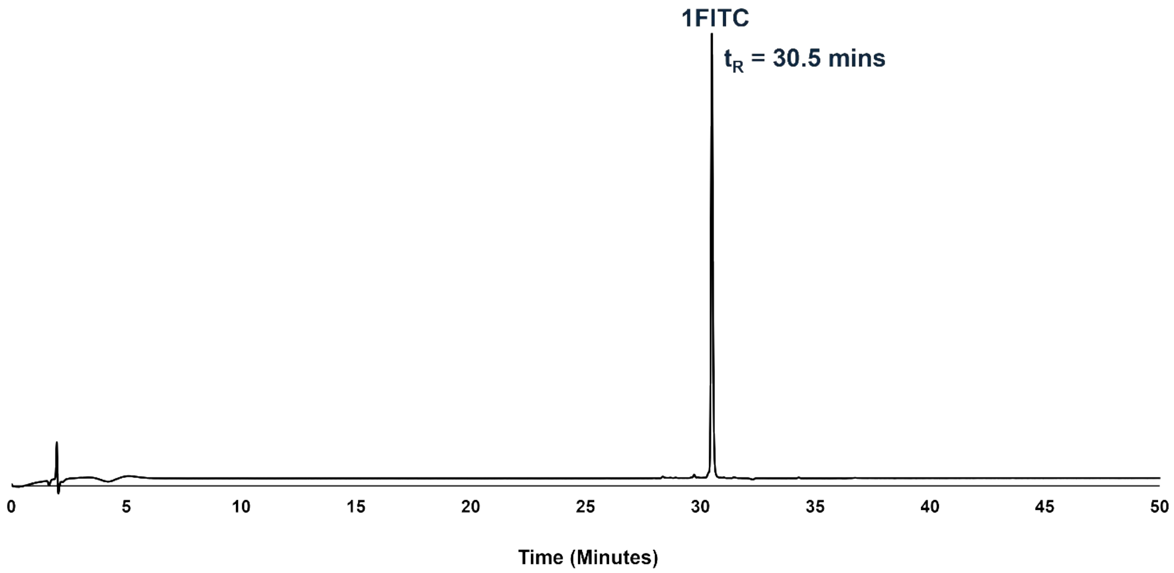

Figure S4. Analytical HPLC chromatogram (214 nm) of purified peptide, **1FITC** (*ca.* > 99% as analysed by peak area. Phenomenex Aeris 5 µm C18 (100Å, 5 µm, 150 × 4.6 mm). Linear gradient 5%B – 95%B over 50 mins (*ca.* 1.8%B/min) at 1 mL/min. t_R = 30.5 mins.

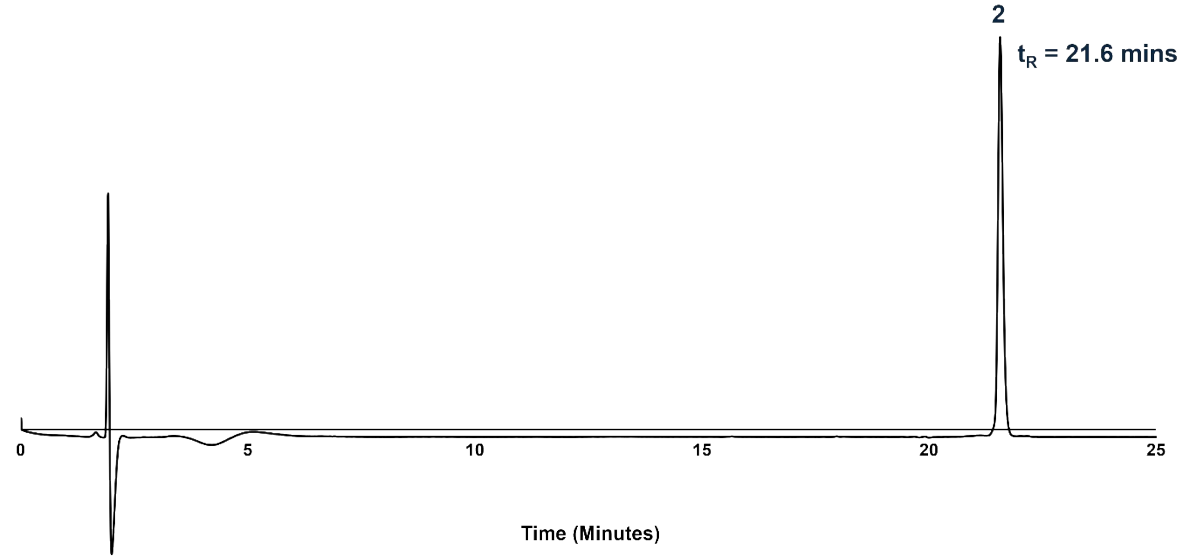

Figure S5. Analytical HPLC chromatogram (214 nm) of purified peptide, **2** (*ca.* > 99% as analysed by peak area. Phenomenex Aeris 5 µm C18 (100Å, 5 µm, 150 × 4.6 mm). Linear gradient 5%B – 95%B over 20 mins (*ca.* 4.5%B/min) at 1 mL/min. t_R = 21.6 mins.

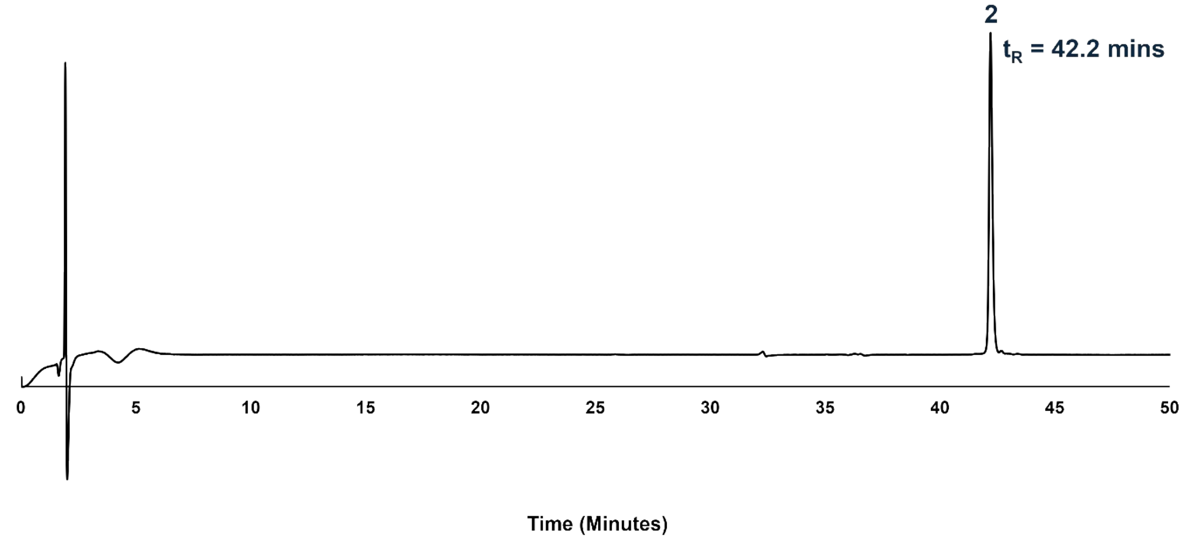

Figure S6. Analytical HPLC chromatogram (214 nm) of purified peptide, **2** (*ca.* > 99% as analysed by peak area. Phenomenex Aeris 5 µm C18 (100Å, 5 µm, 150 × 4.6 mm). Linear gradient 5%B – 95%B over 50 mins (*ca.* 1.8%B/min) at 1 mL/min. t_R = 42.2 mins.

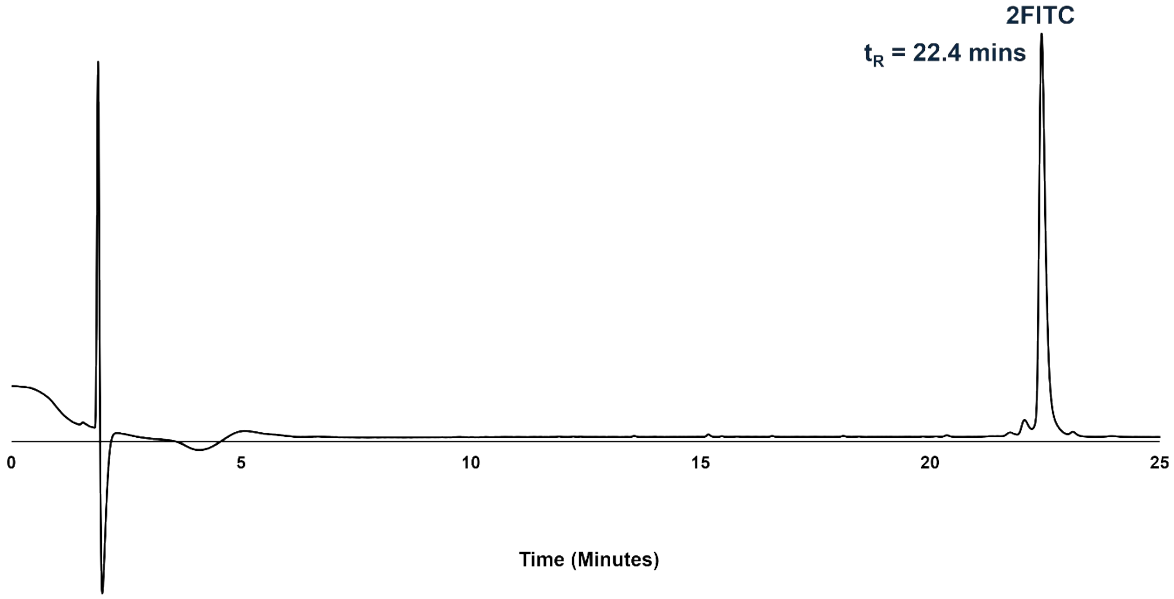

Figure S7. Analytical HPLC chromatogram (214 nm) of purified peptide, **2FITC** (*ca.* > 99% as analysed by peak area. Phenomenex Aeris 5 µm C18 (100Å, 5 µm, 150 × 4.6 mm). Linear gradient 5%B – 95%B over 20 mins (*ca.* 4.5%B/min) at 1 mL/min. t_R = 22.4 mins.

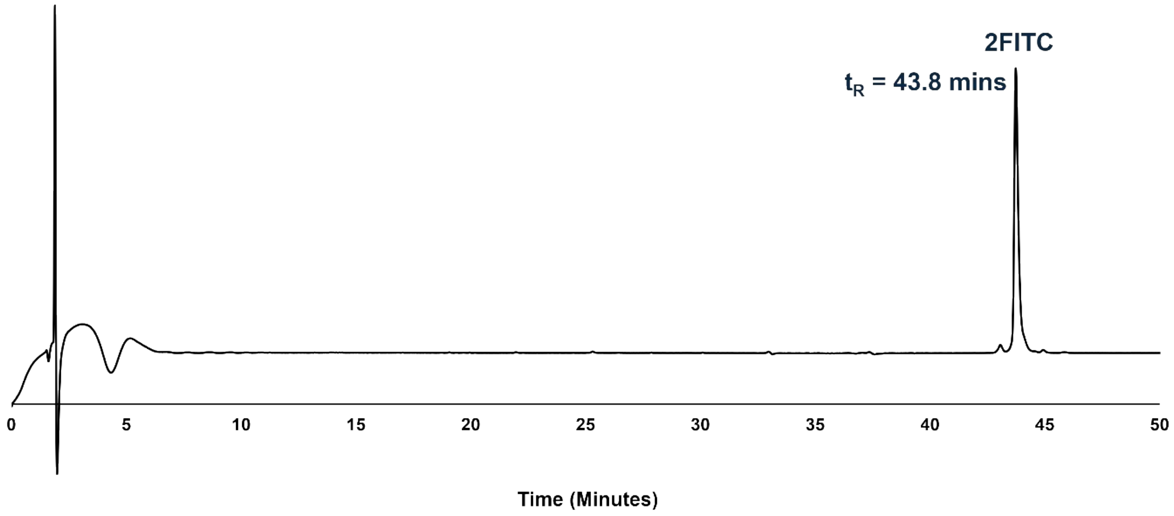

Figure S8. Analytical HPLC chromatogram (214 nm) of purified peptide, **2FITC** (*ca.* > 99% as analysed by peak area. Phenomenex Aeris 5 µm C18 (100Å, 5 µm, 150 × 4.6 mm). Linear gradient 5%B – 95%B over 50 mins (*ca.* 1.8%B/min) at 1 mL/min. t_R = 43.8 mins.

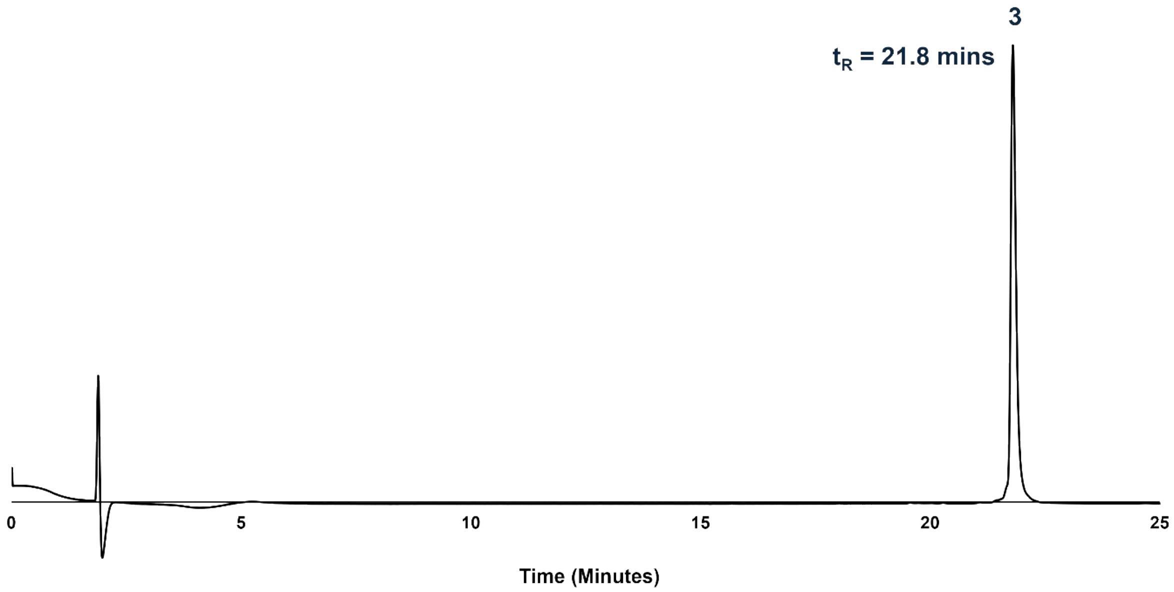

Figure S9. Analytical HPLC chromatogram (214 nm) of purified peptide, **3** (*ca.* > 97% as analysed by peak area. Phenomenex Aeris 5 µm C18 (100Å, 5 µm, 150 × 4.6 mm). Linear gradient 5%B – 95%B over 20 mins (*ca.* 4.5%B/min) at 1 mL/min. t_R = 21.8 mins.

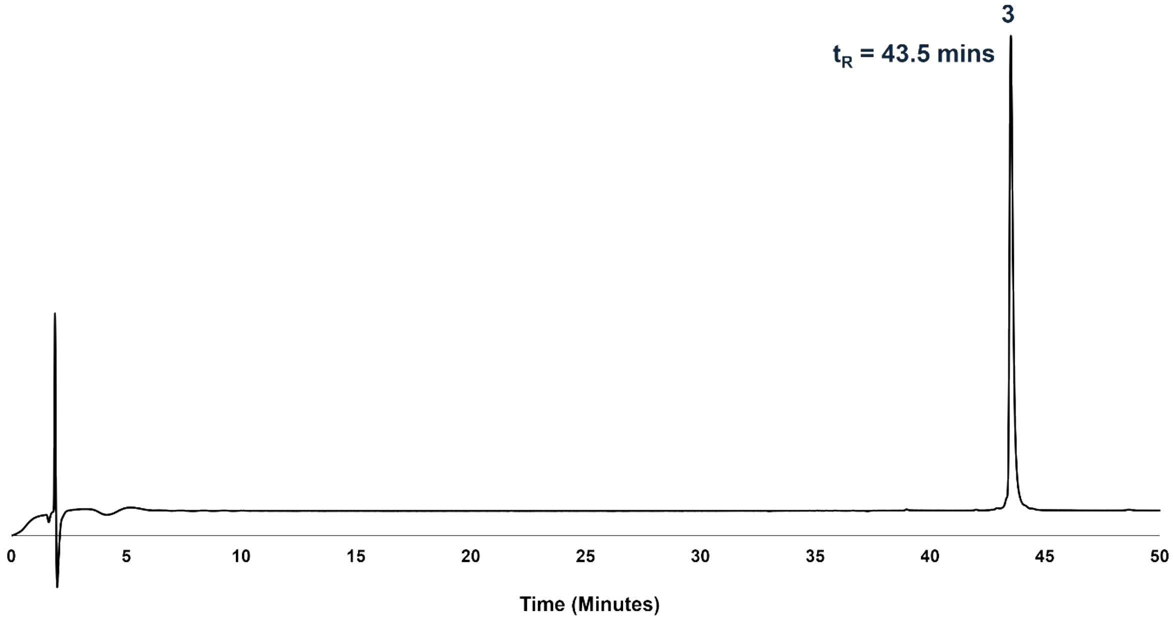

Figure S10 Analytical HPLC chromatogram (214 nm) of purified peptide, **3** (*ca.* > 97% as analysed by peak area. Phenomenex Aeris 5 µm C18 (100Å, 5 µm, 150 × 4.6 mm). Linear gradient 5%B – 95%B over 50 mins (*ca.* 1.8%B/min) at 1 mL/min. t_R = 43.5 mins.

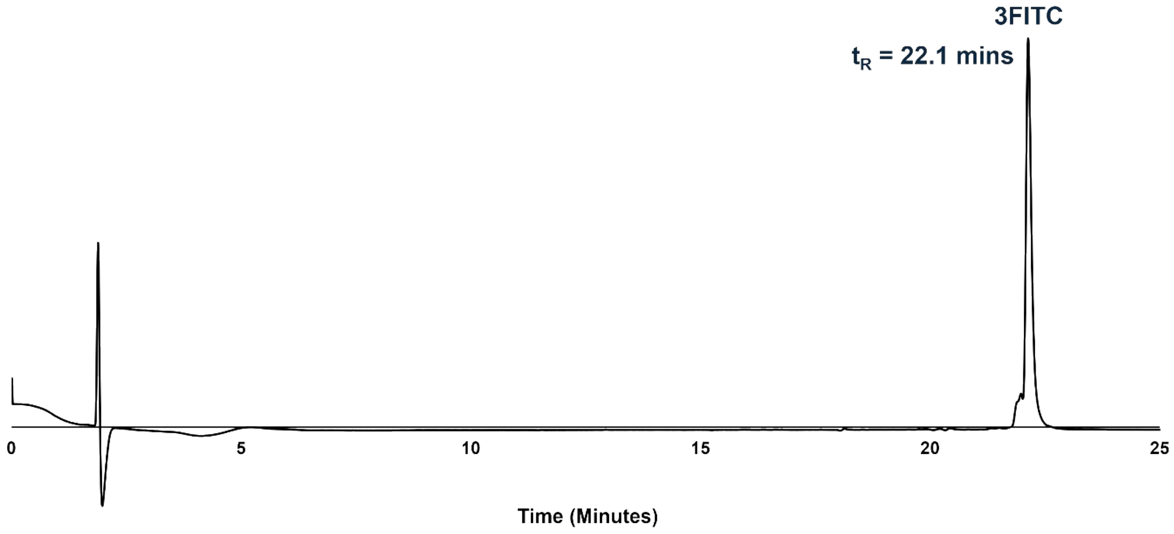

Figure S11. Analytical HPLC chromatogram (214 nm) of purified peptide, **3FITC** (*ca.* > 97% as analysed by peak area. Phenomenex Aeris 5 µm C18 (100Å, 5 µm, 150 × 4.6 mm). Linear gradient 5%B – 95%B over 20 mins (*ca.* 4.5%B/min) at 1 mL/min. t_R = 22.1 mins.

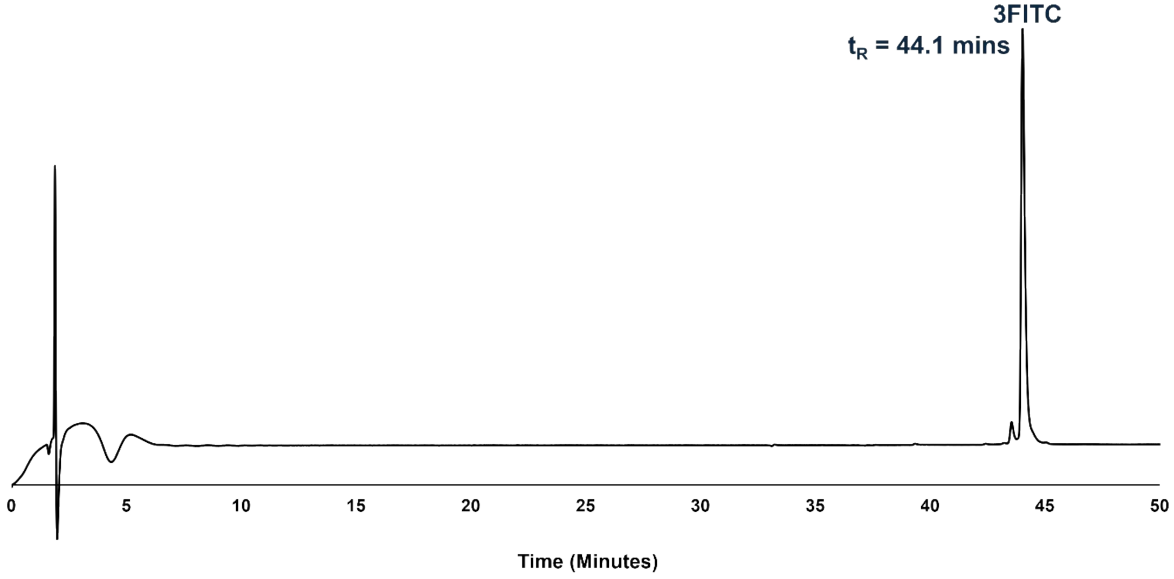

Figure S12. Analytical HPLC chromatogram (214 nm) of purified peptide, **3FITC** (*ca.* > 97% as analysed by peak area. Phenomenex Aeris 5 µm C18 (100Å, 5 µm, 150 × 4.6 mm). Linear gradient 5%B – 95%B over 50 mins (*ca.* 1.8%B/min) at 1 mL/min. t_R = 44.1 mins.

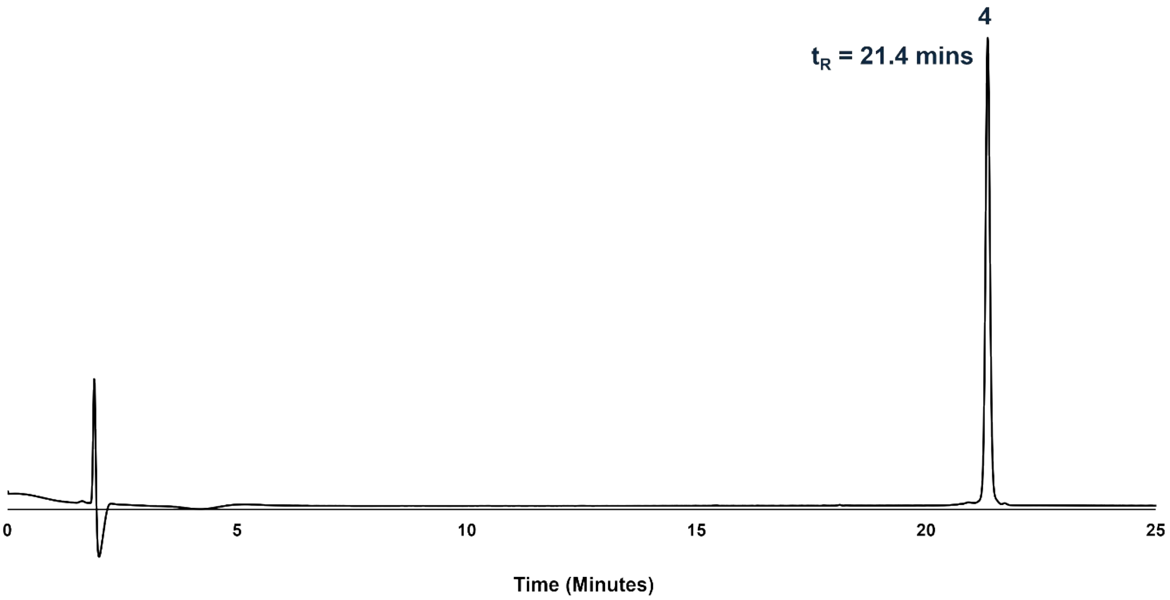

Figure S13. Analytical HPLC chromatogram (214 nm) of purified peptide, **4** (*ca.* > 99% as analysed by peak area. Phenomenex Aeris 5 µm C18 (100Å, 5 µm, 150 × 4.6 mm). Linear gradient 5%B – 95%B over 20 mins (*ca.* 4.5%B/min) at 1 mL/min. t_R = 21.4 mins.

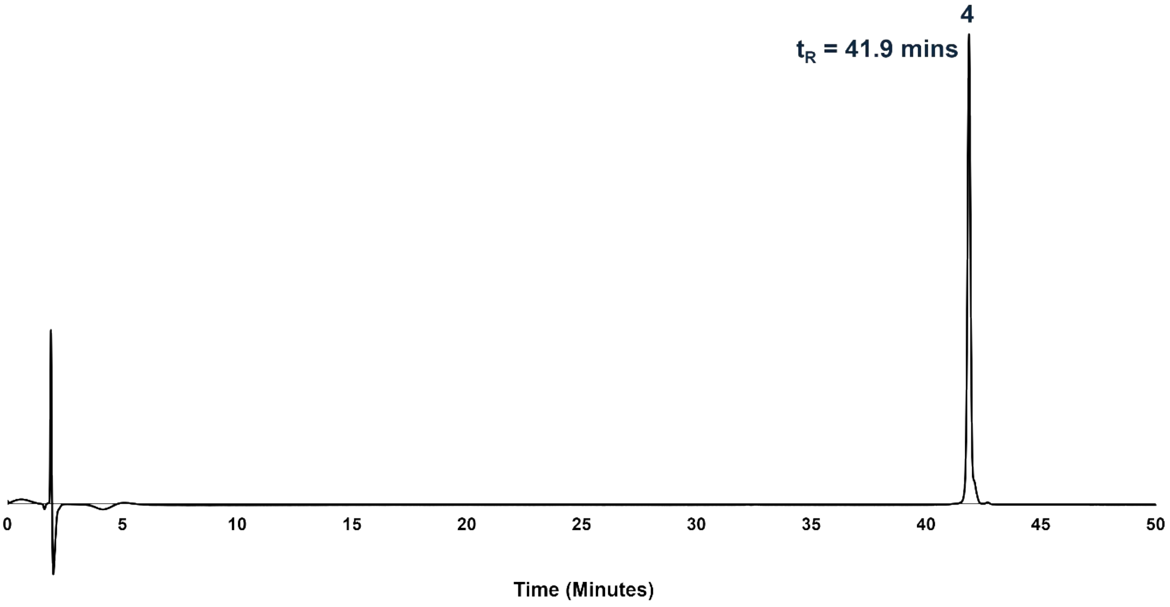

Figure S14. Analytical HPLC chromatogram (214 nm) of purified peptide, **4** (*ca.* > 99% as analysed by peak area. Phenomenex Aeris 5 µm C18 (100Å, 5 µm, 150 × 4.6 mm). Linear gradient 5%B – 95%B over 50 mins (*ca.* 1.8%B/min) at 1 mL/min. t_R = 41.9 mins.

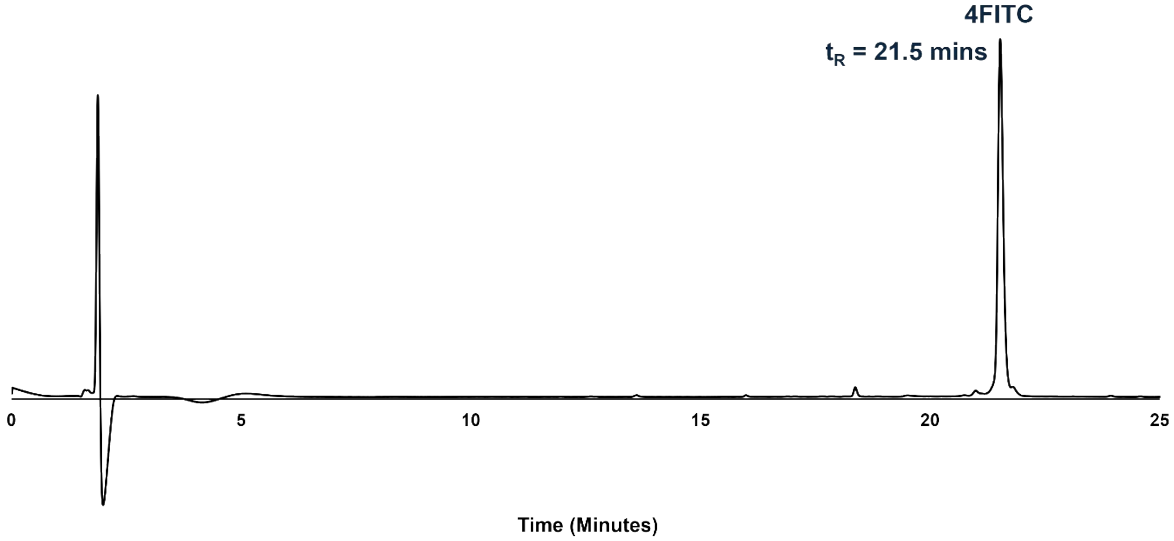

Figure S15. Analytical HPLC chromatogram (214 nm) of purified peptide, **4FITC** (*ca.* > 98% as analysed by peak area. Phenomenex Aeris 5 µm C18 (100Å, 5 µm, 150 × 4.6 mm). Linear gradient 5%B – 95%B over 20 mins (*ca*. 4.5%B/min) at 1 mL/min. t_R = 21.5 mins.

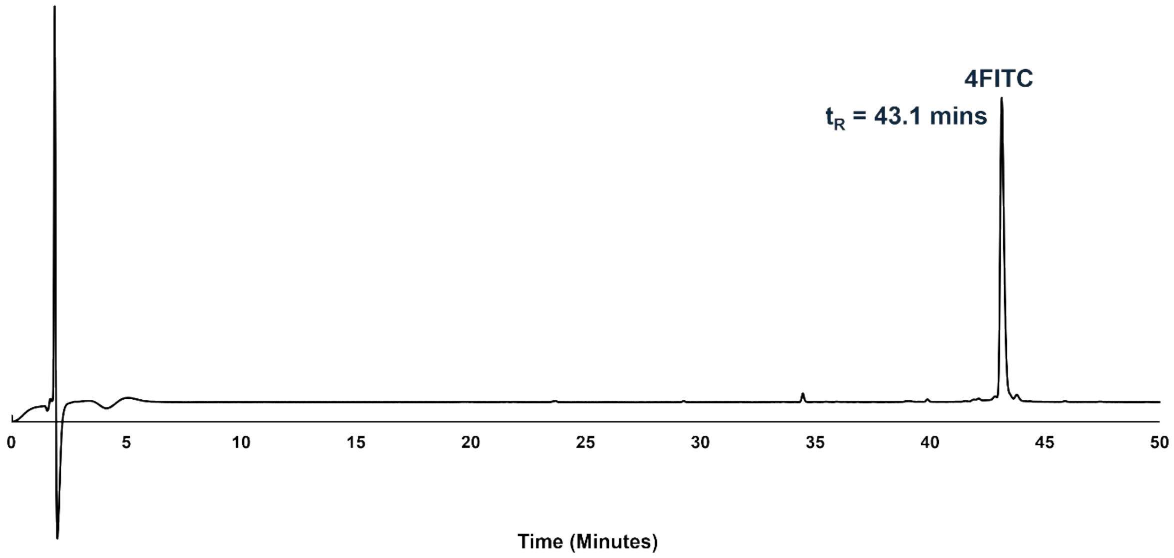

Figure S16. Analytical HPLC chromatogram (214 nm) of purified peptide, **4FITC** (*ca.* > 98% as analysed by peak area. Phenomenex Aeris 5 µm C18 (100Å, 5 µm, 150 × 4.6 mm). Linear gradient 5%B – 95%B over 50 mins (*ca.* 1.8%B/min) at 1 mL/min. t_R = 43.1 mins.

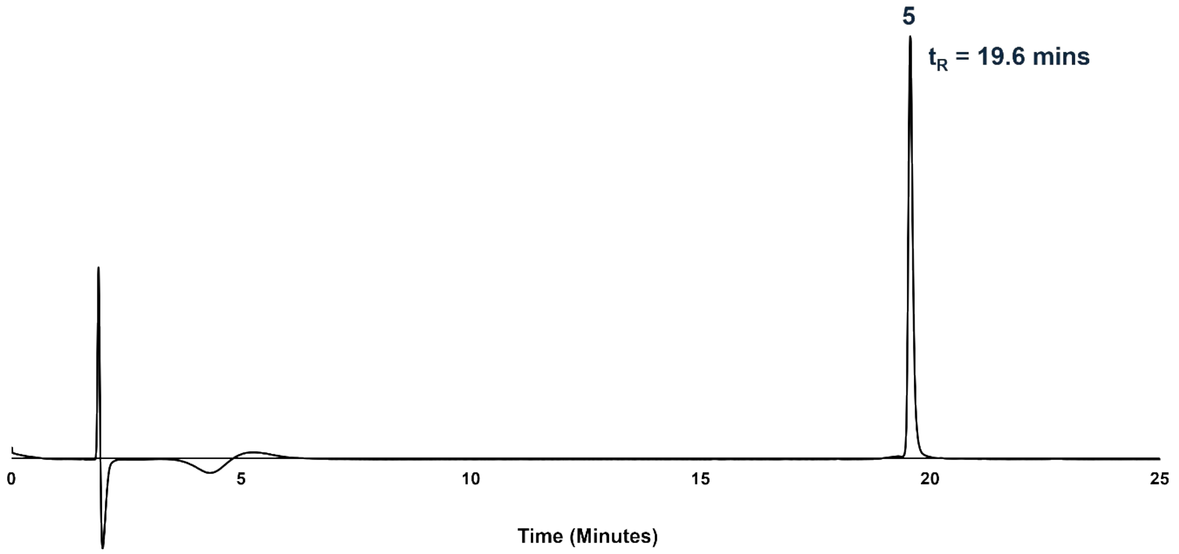

Figure S17. Analytical HPLC chromatogram (214 nm) of purified peptide, **5** (*ca.* > 99% as analysed by peak area. Phenomenex Aeris 5 µm C18 (100Å, 5 µm, 150 × 4.6 mm). Linear gradient 5%B – 95%B over 20 mins (*ca.* 4.5%B/min) at 1 mL/min. t_R = 19.6 mins.

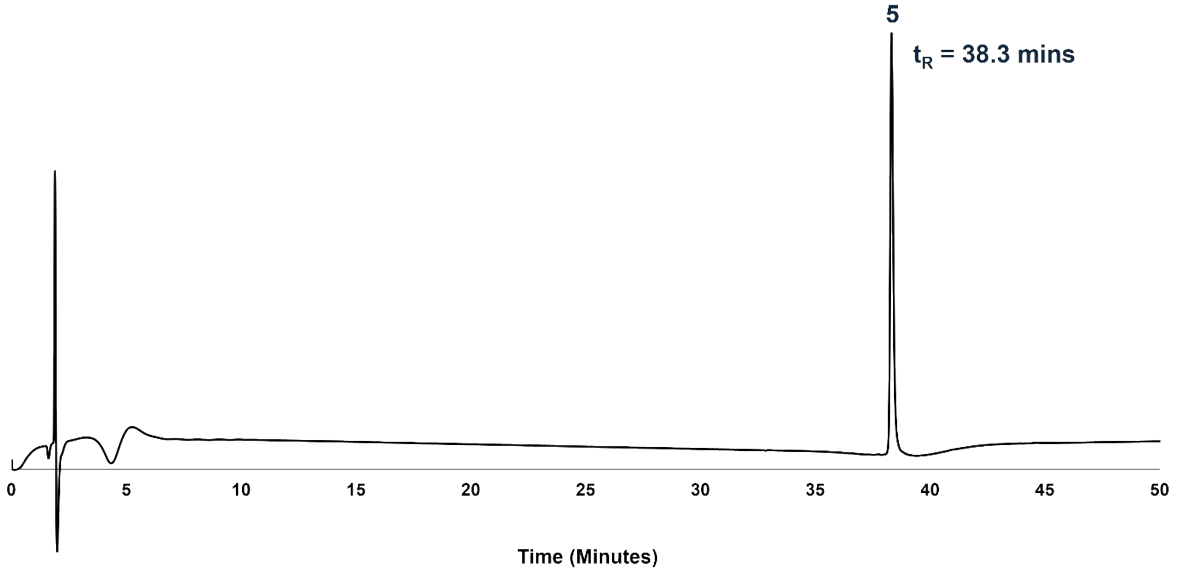

Figure S18. Analytical HPLC chromatogram (214 nm) of purified peptide, **5** (*ca.* > 99% as analysed by peak area. Phenomenex Aeris 5 µm C18 (100Å, 5 µm, 150 × 4.6 mm). Linear gradient 5%B – 95%B over 50 mins (*ca.* 1.8%B/min) at 1 mL/min. t_R = 38.3 mins.

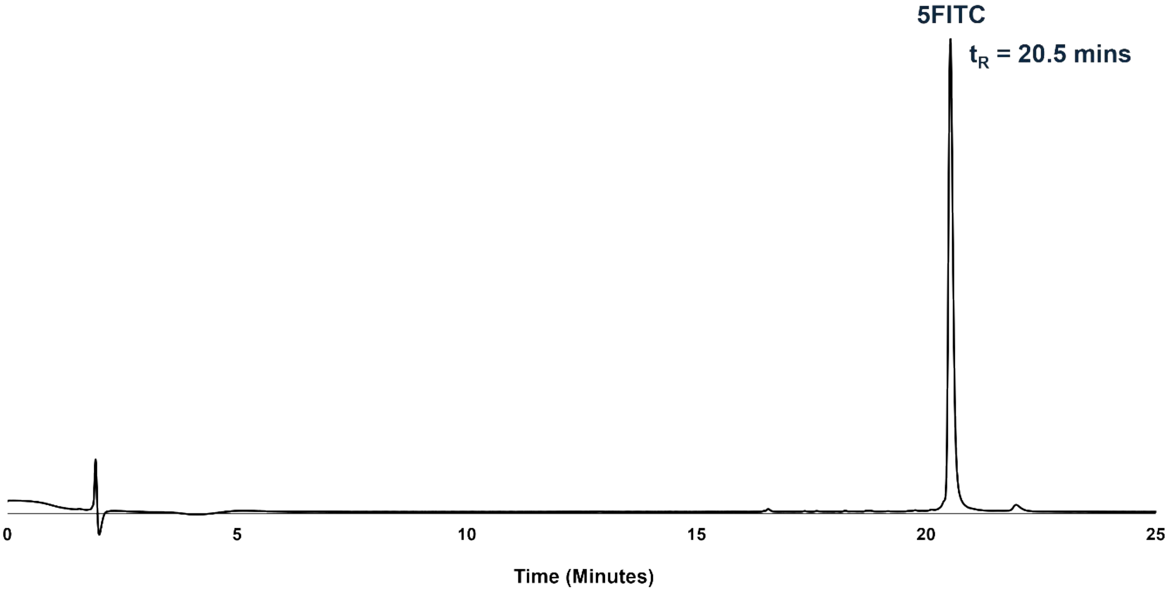

Figure S19. Analytical HPLC chromatogram (214 nm) of purified peptide, **5FITC** (*ca.* > 98% as analysed by peak area. Phenomenex Aeris 5 µm C18 (100Å, 5 µm, 150 × 4.6 mm). Linear gradient 5%B – 95%B over 20 mins (*ca.* 4.5%B/min) at 1 mL/min. t_R = 20.5 mins.

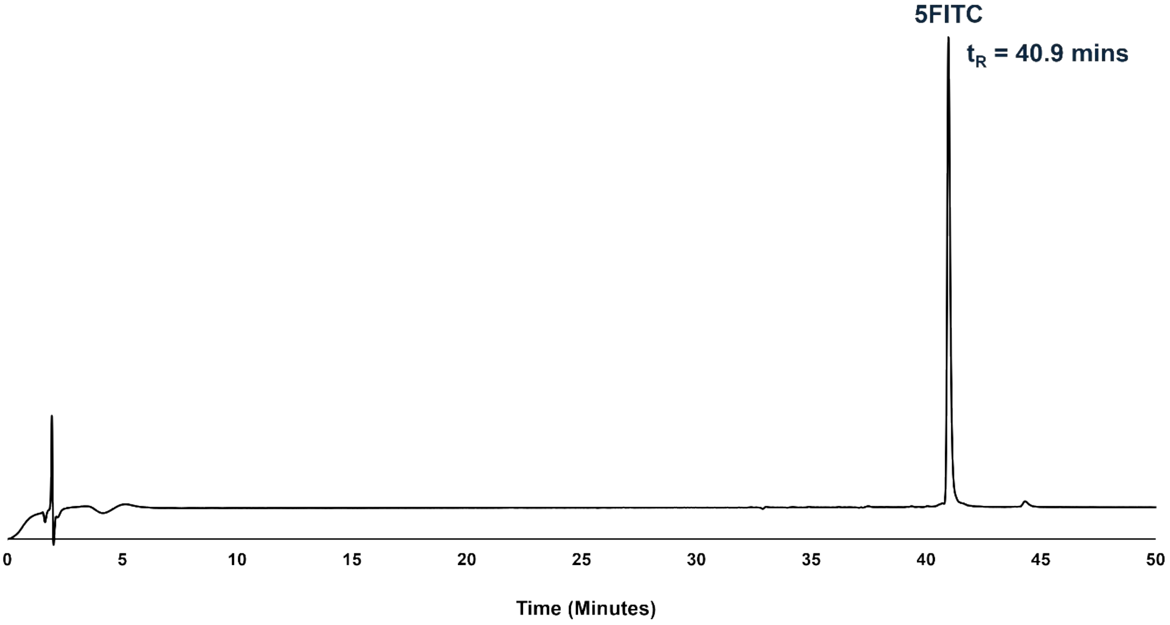

Figure S20. Analytical HPLC chromatogram (214 nm) of purified peptide, **5FITC** (*ca.* > 98% as analysed by peak area. Phenomenex Aeris 5 µm C18 (100Å, 5 µm, 150 × 4.6 mm). Linear gradient 5%B – 95%B over 50 mins (*ca.* 1.8%B/min) at 1 mL/min. t_R = 40.9 mins.

Spectral Data: ESI-MS

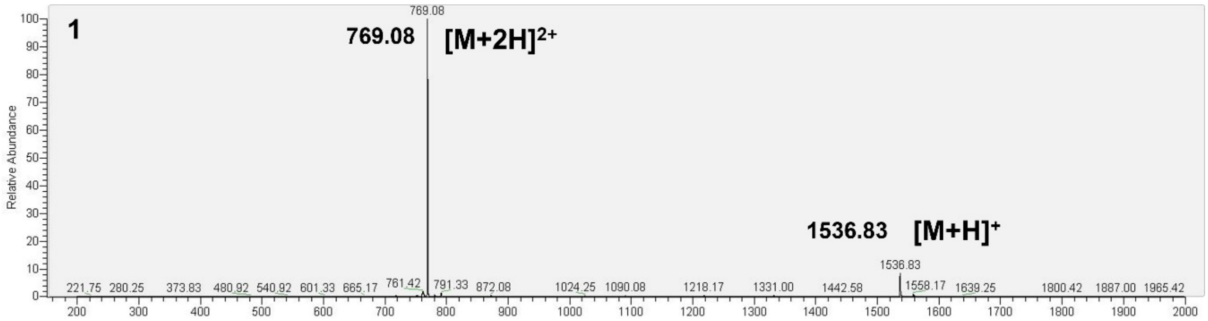

Figure S21. ESI-MS of purified peptide, **1**, *m/z* calculated for [$C_{73}H_{101}N_{17}O_{20}$] 1535.74; deconvoluted mass observed: 1536.00 ± 0.23. Charge states; 769.08 $[M+2H]^{2+}$, 1536.83 $[M+H]^{+}$.

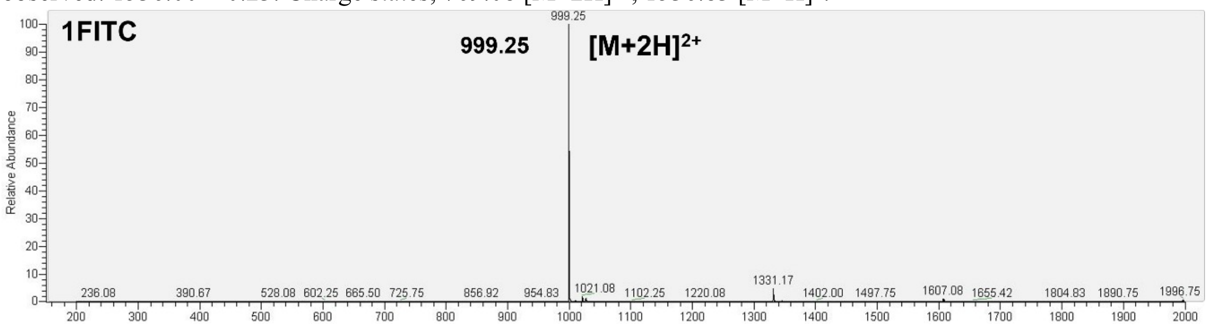

Figure S22. ESI-MS of purified peptide, **1FITC**, *m/z* calculated for [$C_{98}H_{119}N_{19}O_{25}S$ + H] 1996.85; deconvoluted mass observed: 1996.13. Charge state; 999.25 $[M+2H]^{2+}$.

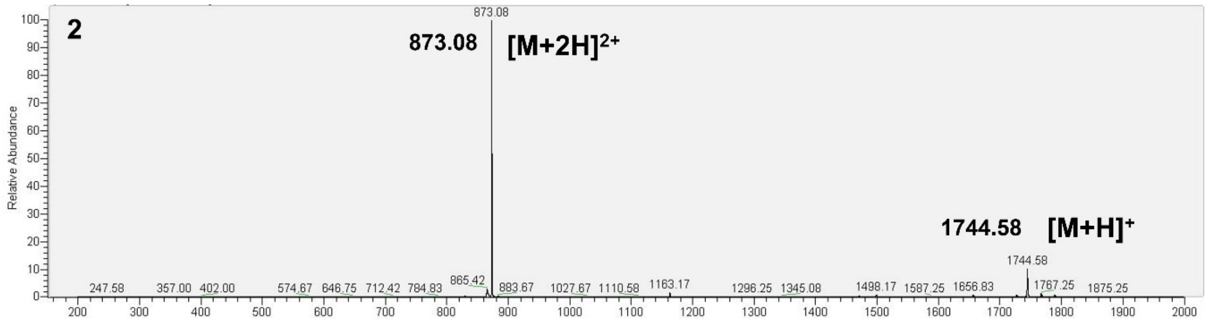

Figure S23. ESI-MS of purified peptide, **2**, *m/z* calculated for [$C_{87}H_{125}N_{17}O_{21}$] 1743.92; deconvoluted mass observed: 1743.87 ± 0.41. Charge states; 873.08 $[M+2H]^{2+}$, 1744.58 $[M+H]^{+}$.

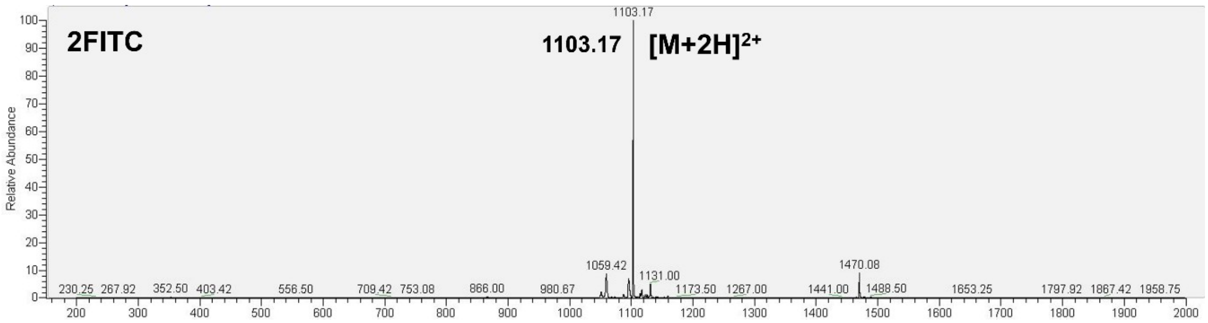

Figure S24. ESI-MS of purified peptide, **2FITC**, *m/z* calculated for [$C_{112}H_{145}N_{19}O_{26}S$] 2205.04; deconvoluted mass observed: 2204.34. Charge state; 1103.17 $[M+2H]^{2+}$.

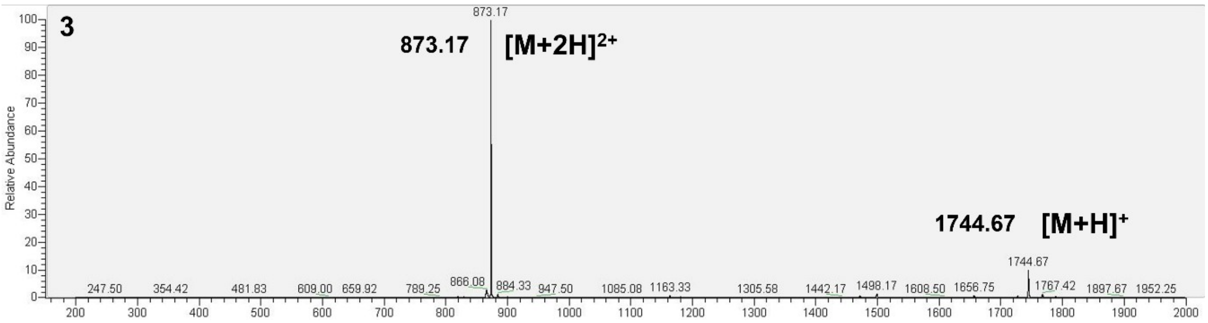

Figure S25. ESI-MS of purified peptide, **3**, *m/z* calculated for [$C_{87}H_{125}N_{17}O_{21}$] 1743.92; deconvoluted mass observed: 1744.01 ± 0.47. Charge states; 873.17 $[M+2H]^{2+}$, 1744.67 $[M+H]^{+}$.

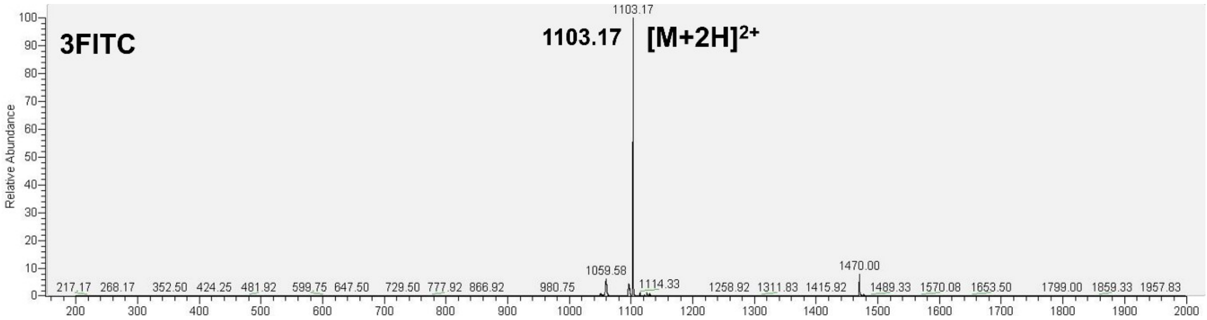

Figure S26. ESI-MS of purified peptide, **3FITC**, *m/z* calculated for [$C_{112}H_{145}N_{19}O_{26}S$] 2205.04; deconvoluted mass observed: 2204.34. Charge state; 1103.17 $[M+2H]^{2+}$.

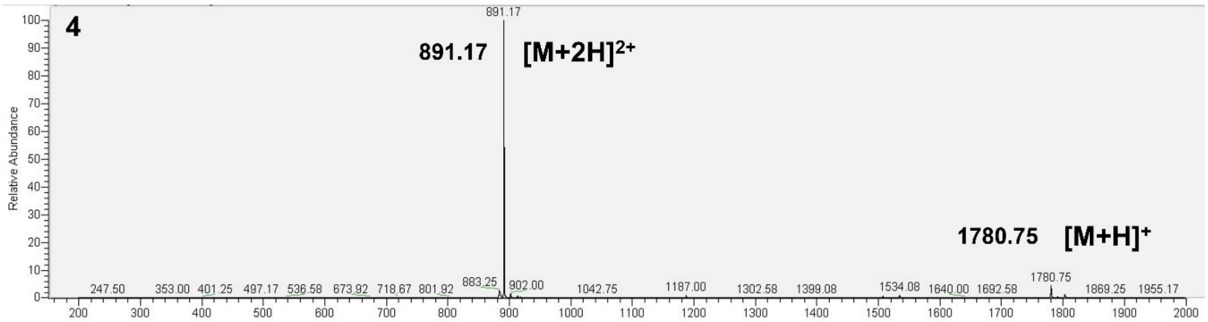

Figure S27. ESI-MS of purified peptide, **4**, *m/z* calculated for [$C_{90}H_{125}N_{17}O_{21}$] 1779.92; deconvoluted mass observed: 1780.05 ± 0.42. Charge states; 891.17 $[M+2H]^{2+}$, 1780.75 $[M+H]^{+}$.

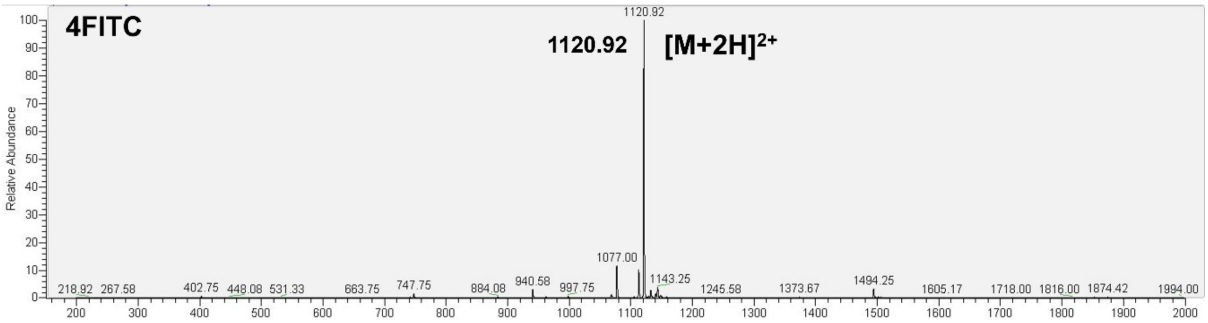

Figure S28. ESI-MS of purified peptide, **4FITC**, *m/z* calculated for [$C_{115}H_{146}N_{19}O_{26}S$] 2240.04; deconvoluted mass observed: 2239.84. Charge state; 1120.92 $[M+2H]^{2+}$.

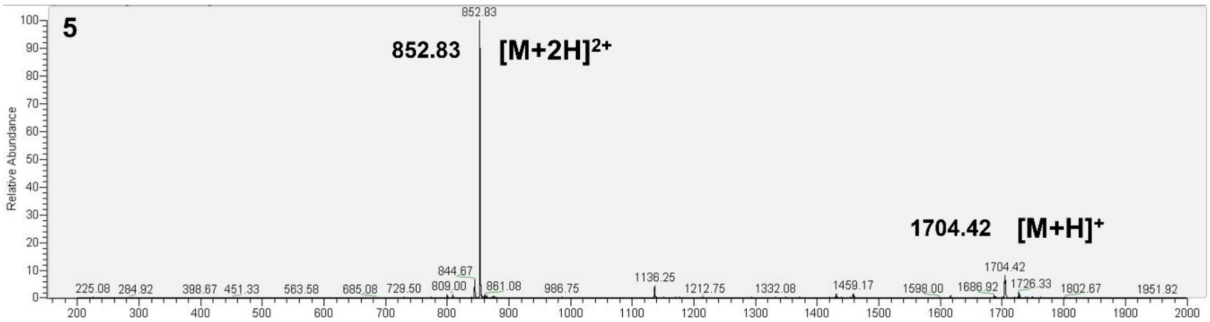

Figure S29. ESI-MS of purified peptide, **5**, *m/z* calculated for [$C_{84}H_{121}N_{17}O_{21}$] 1703.89; deconvoluted mass observed: 1703.54 ± 0.17. Charge states; 852.83 $[M+2H]^{2+}$, 1704.42 $[M+H]^{+}$.

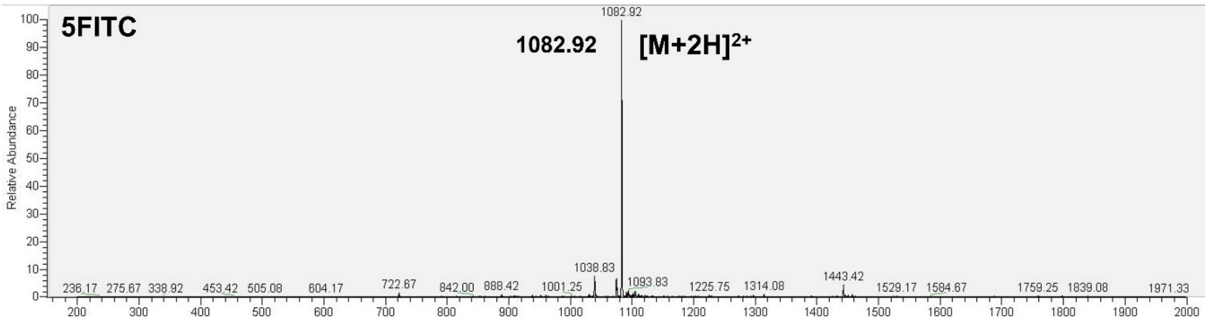

Figure S30. ESI-MS of purified peptide, **5FITC**, *m/z* calculated for [$C_{109}H_{141}N_{19}O_{26}S$] 2165.00; deconvoluted mass observed: 2163.84. Charge state; 1082.92 $[M+2H]^{2+}$.

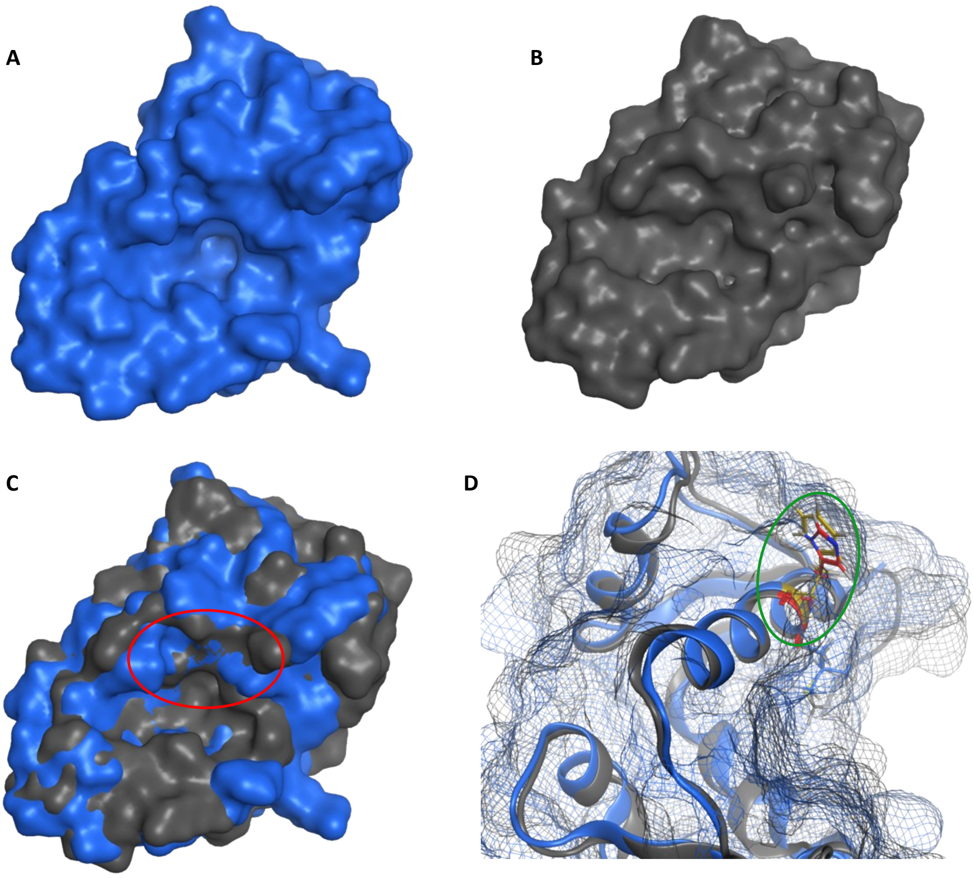

Supporting Information Tables and Figure

Figure S31. Molecular models of (**A**) MDM2 (blue) and (**B**) MDMX (grey). (**C**) Overlapped MDM2 and MDMX, the red oval highlights the proposed binding pocket for the stapled peptides. (**D**) A zoomed view with green oval of the binding pocket revealing key residues influencing the shape of the pocket (Phe_{55} of MDM2 in yellow, and His_{55} of MDMX in red).

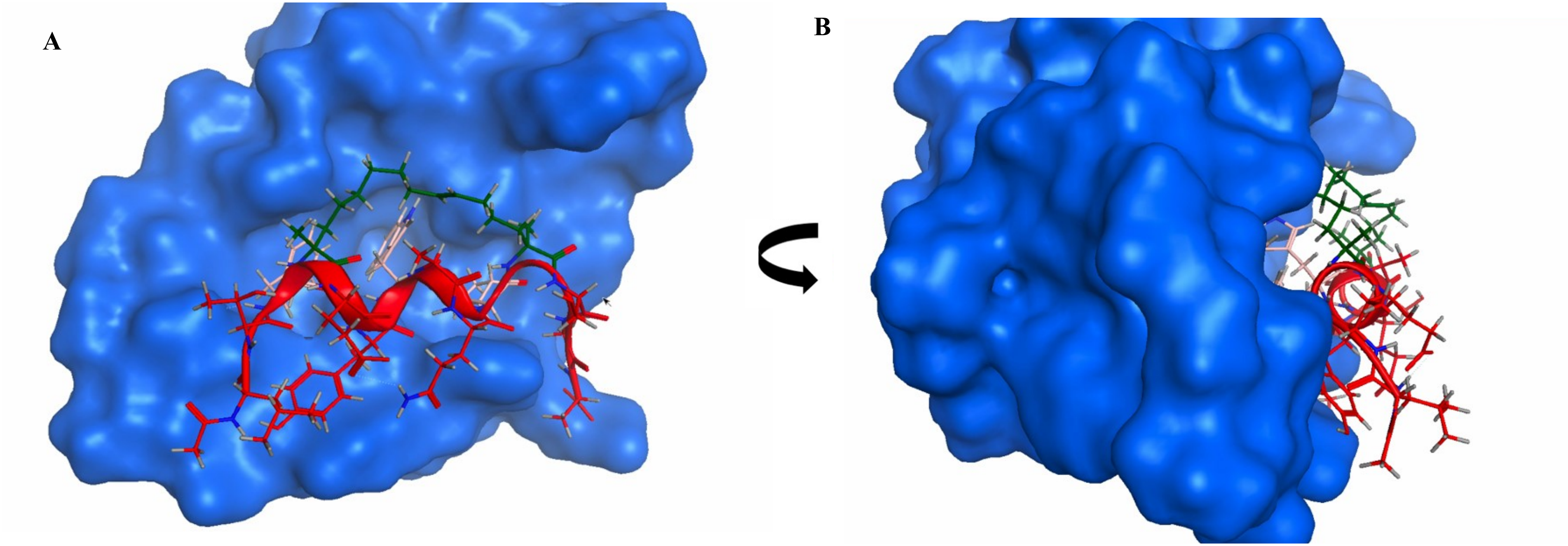

Pose	PDB	S
**1**	1YCR	-10.9718
**2**	1YCR	-10.8321
**3**	1YCR	-10.8276
**4**	1YCR	-9.9695
**5**	1YCR	-9.9139

Figure S32. MOE docking experiments showing the binding of ATSP-7041 to MDM2 (blue). (**A**) Front view. (**B**) Side view. The hydrocarbon staple (green) fits well into the binding pocket with the key interacting residues of the peptide being Phe, Trp and Cba (pink). **Table:** Binding data from the docking experiment gave favourable MOE docking scores (negative values) for the top hits. S = MOE docking score.

A

B

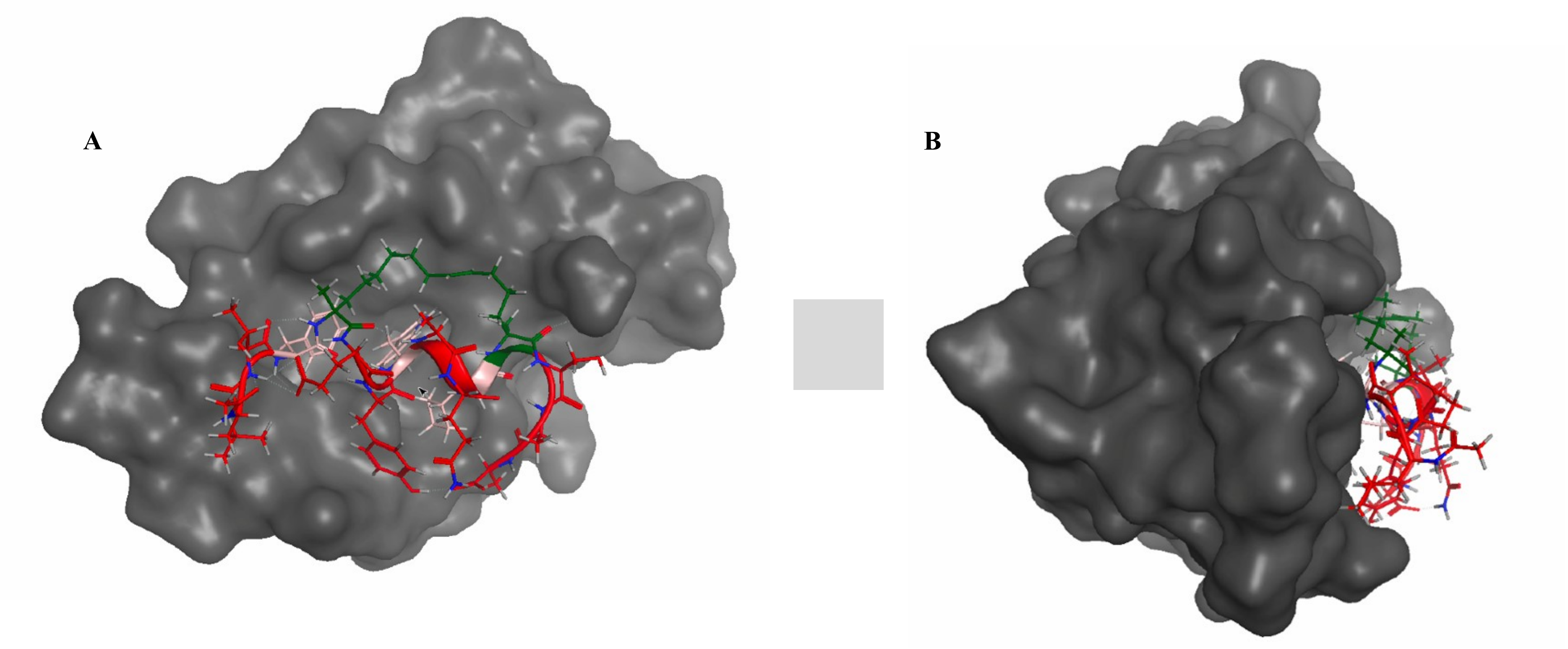

Pose	PDB	S
**1**	4N5T	-11.2922
**2**	4N5T	-11.1267
**3**	4N5T	-11.0198
**4**	4N5T	-10.0661
**5**	4N5T	-8.4940

Figure S33. MOE docking experiments showing the binding of ATSP-7041 to MDMX (grey). (**A**) Front view. (**B**) Side view. The hydrocarbon staple (green) fits tightly into the binding pocket with the key interacting residues of the peptide being Phe, Trp and Cba (pink). **Table:** Binding data from the docking experiment gave favourable MOE docking scores (negative values) for the top hits. S = MOE docking score.

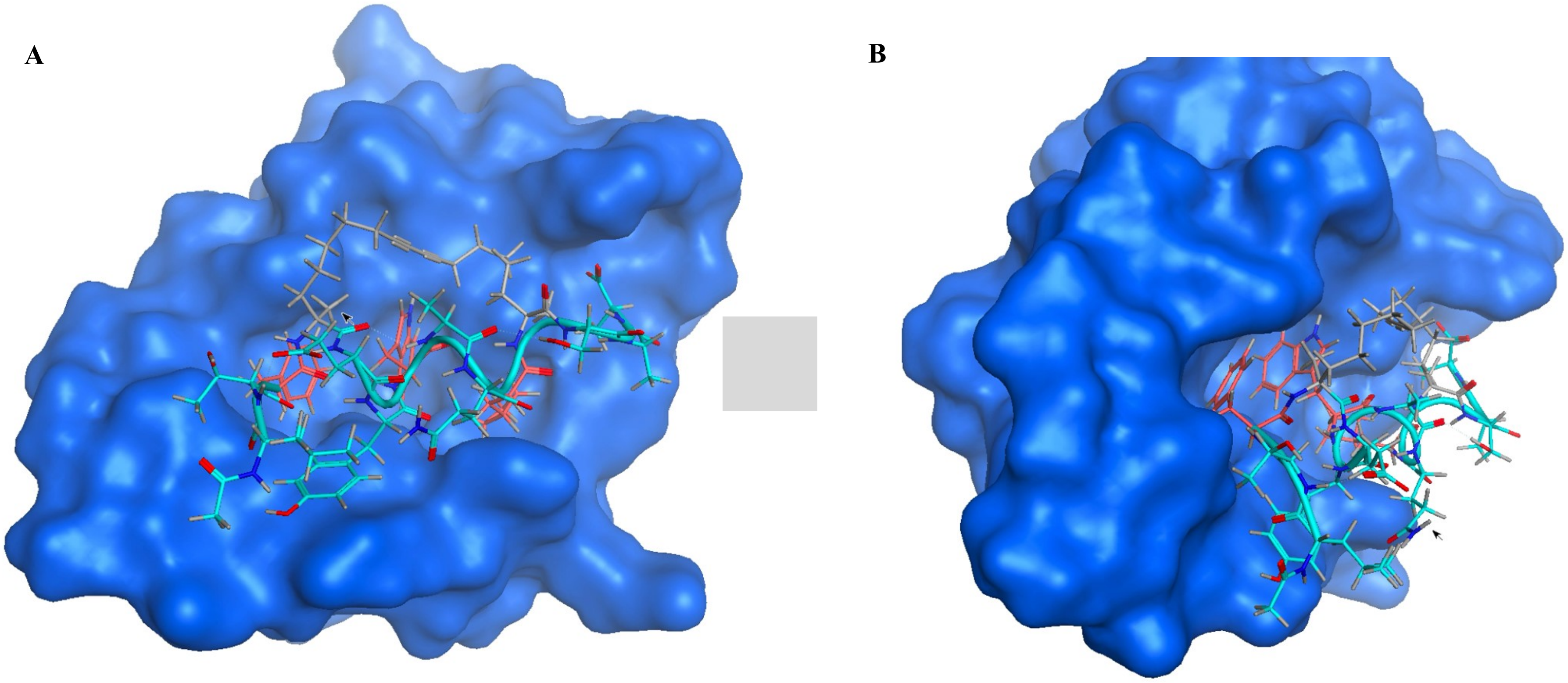

Pose	PDB	S
**1**	1YCR	-11.2507
**2**	1YCR	-11.1911
**3**	1YCR	-10.5301
**4**	1YCR	-10.3677
**5**	1YCR	-10.3443

Figure S34. MOE docking experiments showing the bi
peptide to MDM2 (blue). (**A**) Front view. (**B**) Side view.
the binding pocket with the key interacting residues of
and Cba (pink). **Table:** Binding data from the docking e
MOE docking scores (negative values) for the top hits. S

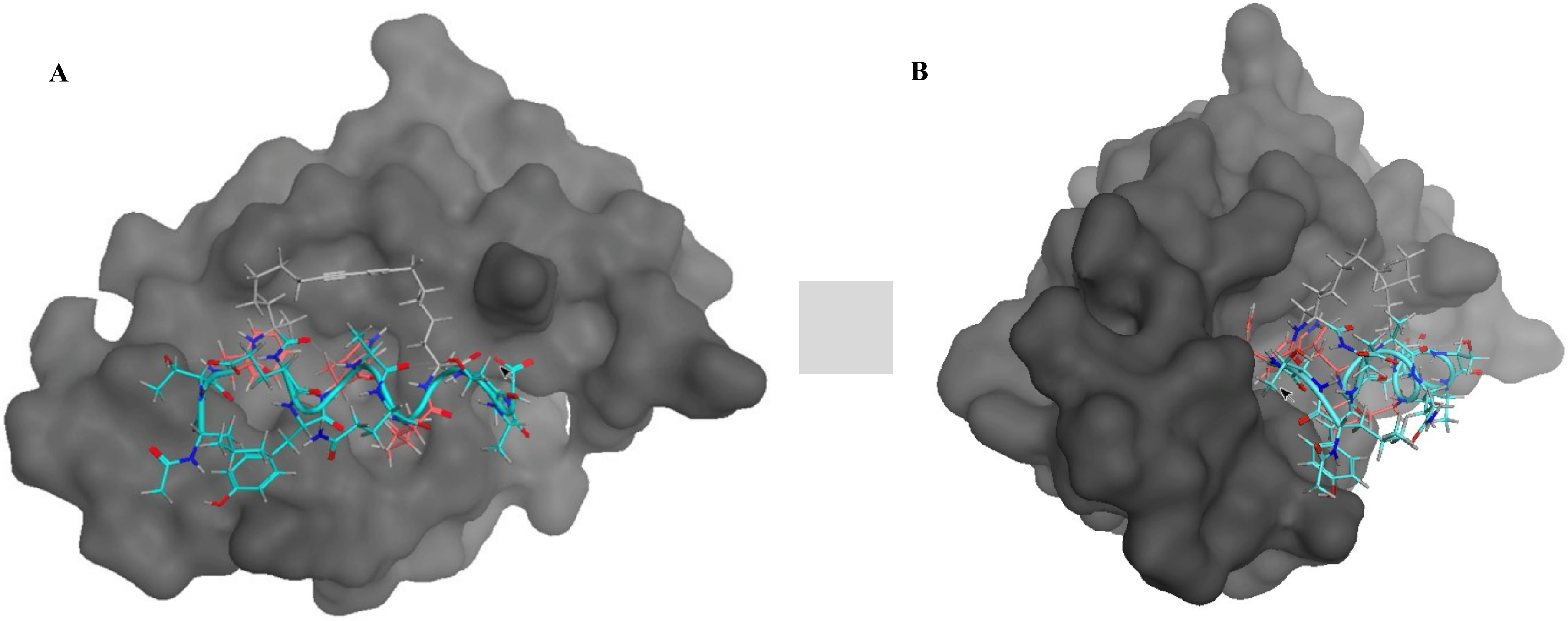

Pose	PDB	S
**1**	4N5T	-11.7590
**2**	4N5T	93.7123
**3**	4N5T	240.7505
**4**	4N5T	240.7507
**5**	4N5T	242.5198

Figure S35. MOE docking experiments showing the binding of the diyne stapled peptide to MDMX (grey). (**A**) Front view. (**B**) Side view. The key interacting residues of the peptide being Phe, Trp and Cba (pink). **Table:** Binding data from the docking experiment gave mainly unfavourable MOE docking scores (positive values). The scores indicated that only one conformation is favourable for binding. S = MOE docking score.

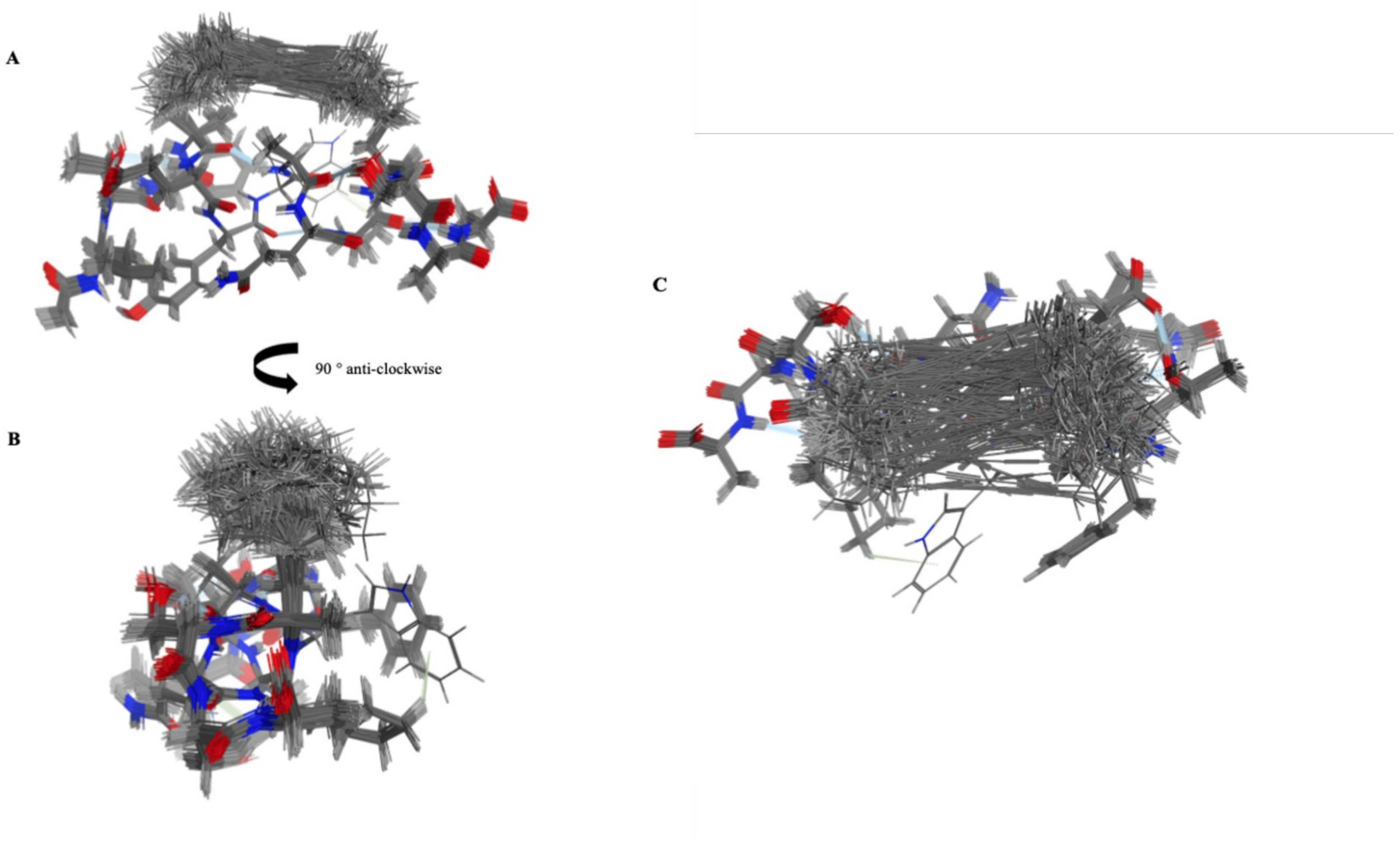

Figure S36. A conformational search was conducted on the diyne stapled peptide using MOE, to predict the conformational variability. The most conformationally variability occurs in the staple region. (**A**) Front-view showing the overlap of the amino acid regions. (**B**) Side-view again showing the overlap of the amino acid regions and the Trp reference. (**C**) Top-view showing the variability of the diyne staple

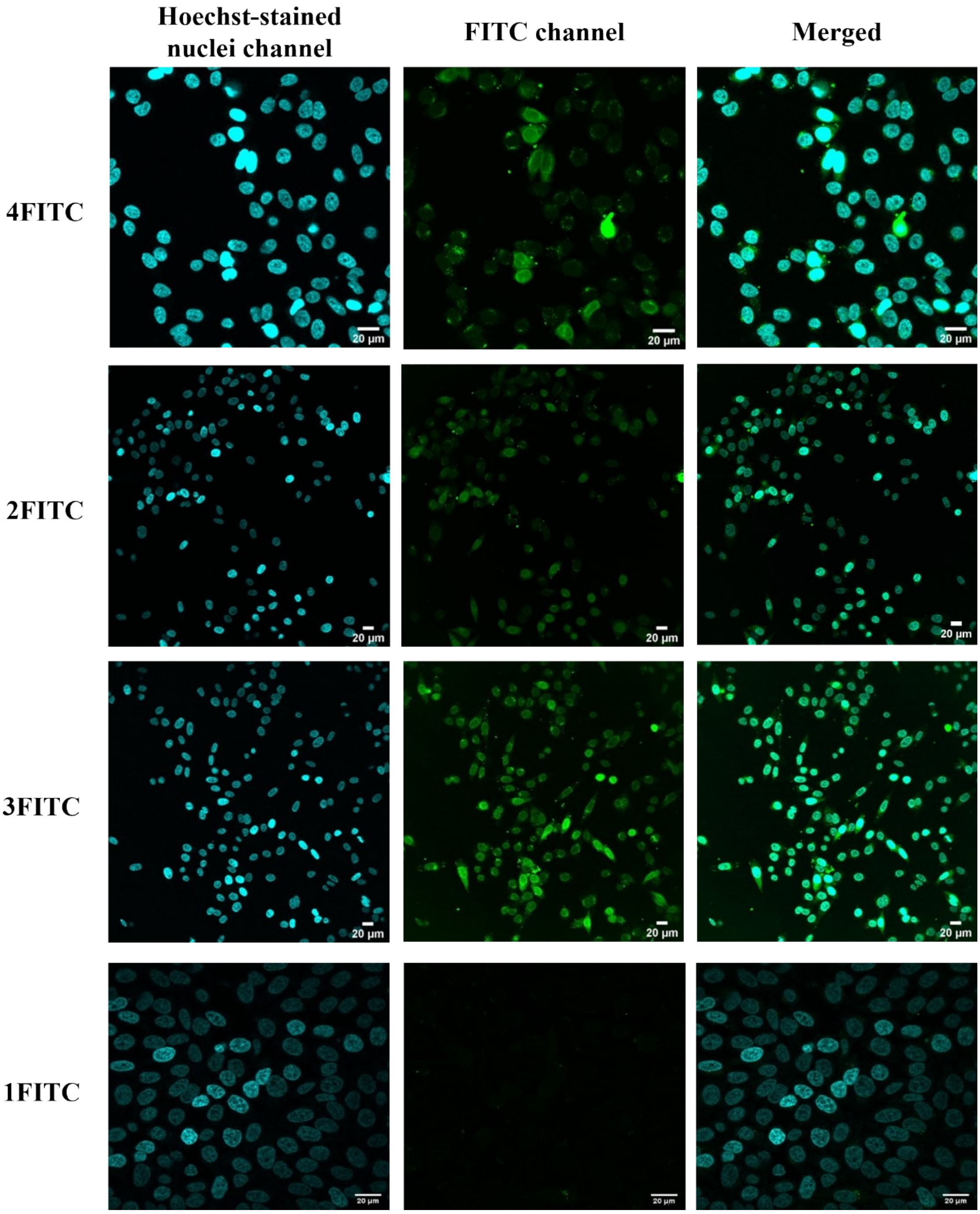

Figure S37. Fluorescence imaging showing the fluorescein-labelled diyne stapled peptide 4FITC and alkene stapled peptides 2FITC/3FITC internalised in HeLa cells. The native peptide 1FITC showed no internalisation. The cells were stained with Hoechst, a nuclear stain. Left lane: Hoechst-stained nuclei channel. Middle: FITC channel. Right: Merged cell image. Microscopy images were acquired with a custom-built multi-modal microscope setup. Images were processed using ImageJ 1.53c, scale bar = 20 µm.

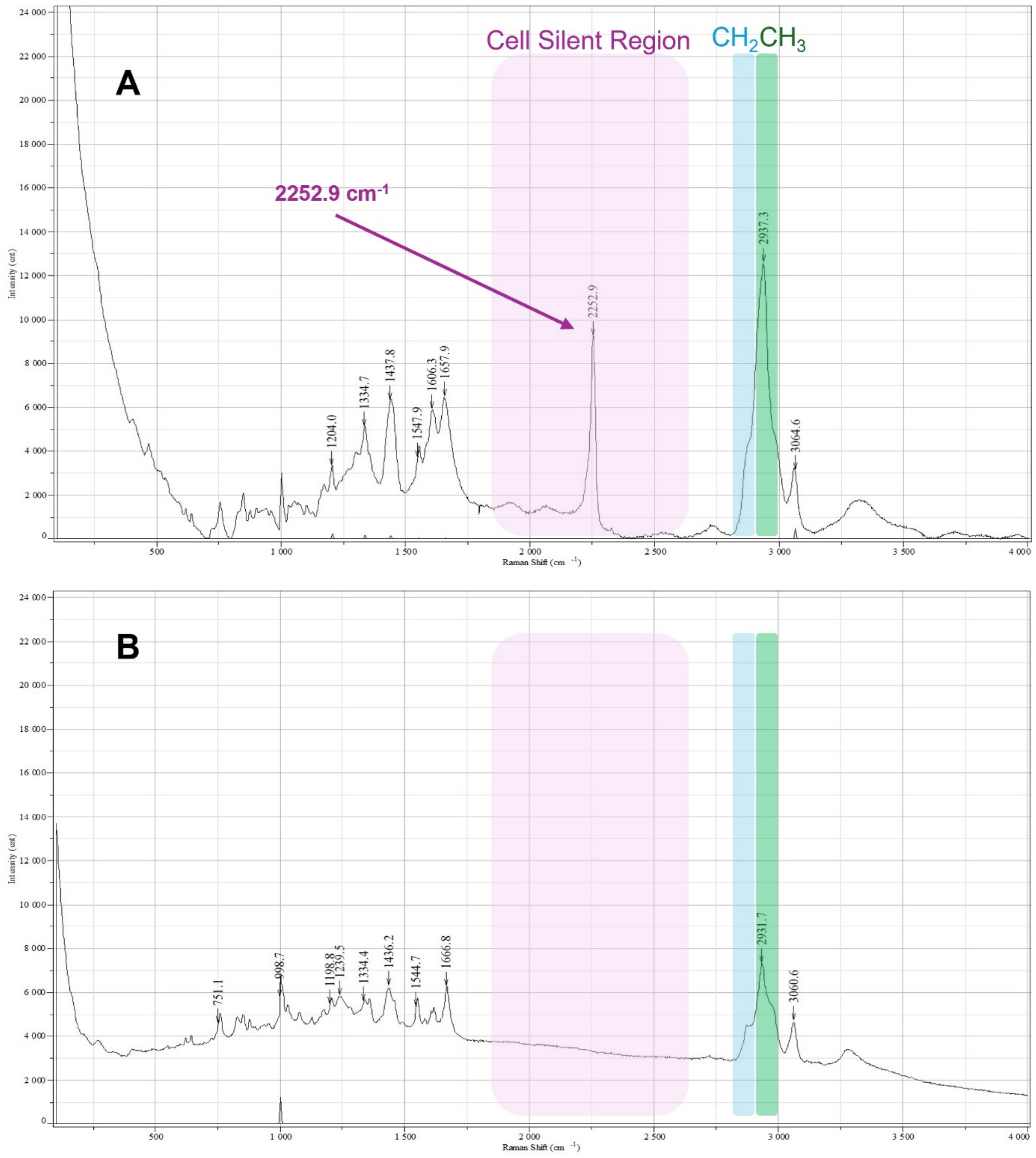

Figure S38. Solid State Raman Spectroscopy of peptides; A) diyne **4** and B) *trans*-alkene, **3**.

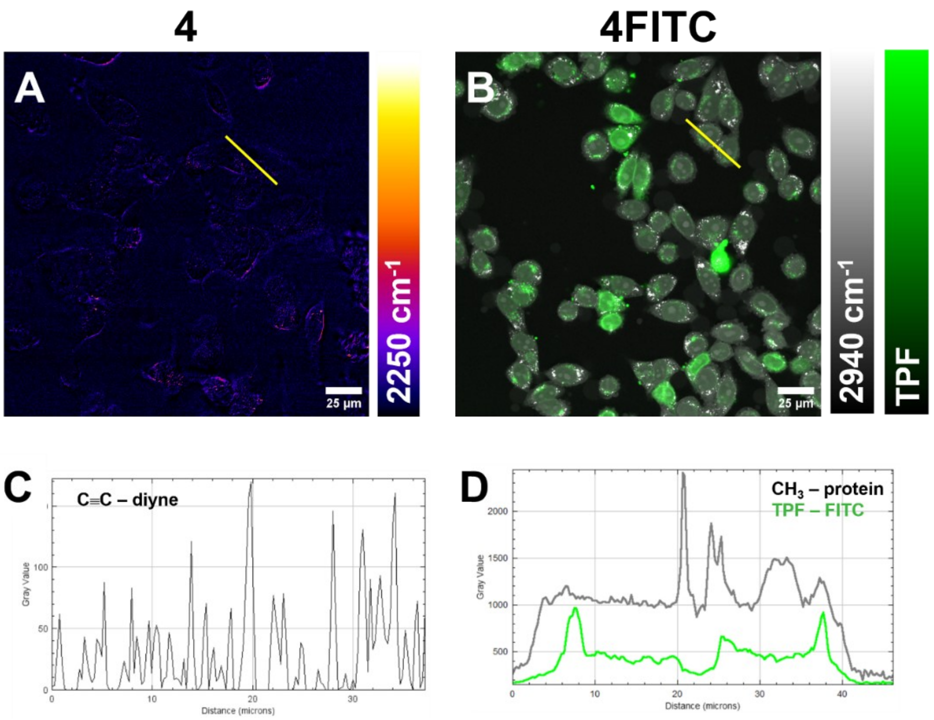

Figure S39. Multimodal imaging of **4** and **4FITC** in live cells. A) ES-2 cells were incubated with **4** (100 µM, 3 h); B) HeLa cells were incubated with **4FITC** (20 µM, 3 h). C/D) show signal intensity along the region selected for signal measurement (yellow line in A and B). Contrast achieved by tuning to: A) SRS 2250 cm^{-1}, alkyne (stapled peptide); B) Overlay of SRS 2940 cm^{-1}, CH_3 (proteins) and TPF signal from FITC. C) stapled peptide intensity (**4**, grey); and D) CH_3 intensity (cellular proteins, grey) and FITC intensity (**4FITC**, green). Microscopy images were acquired with a custom-built multi-modal microscope setup. Alkyne image background subtracted using off-resonance image and modified with false colouring for clarity. Images were processed using ImageJ 1.53c, scale bar = 25 µm.

References

1. Luo, P.; Baldwin, R. L. Mechanism of Helix Induction by Trifluoroethanol: A Framework for Extrapolating the Helix-Forming Properties of Peptides from Trifluoroethanol/Water Mixtures Back to Water. *Biochemistry* **1997**, *36* (27), 8413–8421. https://doi.org/10.1021/bi9707133.

2. Johnson Jr, W. C.; Tinoco Jr, I. Circular Dichroism of Polypeptide Solutions in the Vacuum Ultraviolet. *Journal of the American Chemical Society* **1972**, *94* (12), 4389–4390.

3. Steven, C.F., Lee, M., Nichol, G.S., Davey, P.R., Chiarparin, E., Brunton, V.G. and Hulme, A.N., 2022. Design, Synthesis, and Analytical Evaluation of Fsp3-Inspired Raman Probes for Cellular Imaging. *European Journal of Organic Chemistry*, **2022**, (30), p.e202200393.

4. Studier, F. W. Protein production by auto-induction in high-density shaking cultures. *Protein expression and purification* **2005**, *41*(1), 207-234.

5. Morgan, D. C.; McDougall, L.; Knuhtsen, A.; Jamieson, A. G. Development of Bifunctional, Raman Active Diyne-Girder Stapled α-Helical Peptides. *Chemistry – A European Journal* **2023**, *29* (41), e202300855. https://doi.org/10.1002/chem.202300855.
